# Protein manipulation using single copies of short peptide tags in cultured cells and in *Drosophila melanogaster*

**DOI:** 10.1242/dev.191700

**Published:** 2021-03-16

**Authors:** M. Alessandra Vigano, Clara-Maria Ell, Manuela M. M. Kustermann, Gustavo Aguilar, Shinya Matsuda, Ning Zhao, Timothy J. Stasevich, Markus Affolter, George Pyrowolakis

**Affiliations:** 1 Growth and Development, Biozentrum, University of Basel, Klingelbergstrasse 70, CH-4056 Basel, Switzerland; 2 Spemann Graduate School of Biology and Medicine (SGBM), University of Freiburg, 79104 Freiburg, Germany; 3 Institute for Biology I, Faculty of Biology, University of Freiburg, 79104 Freiburg, Germany; 4 CIBSS – Centre for Integrative Biological Signalling Studies, University of Freiburg, 79104 Freiburg, Germany; 5 Center for Biological Systems Analysis, University of Freiburg, Habsburgerstrasse 49, 79104 Freiburg, Germany; 6 Department of Biochemistry and Molecular Biology, Colorado State University, Fort Collins, CO 80523, USA

**Keywords:** *In vivo*, Nanobodies, Peptide binders, Protein manipulation, Small tag

## Abstract

Cellular development and function rely on highly dynamic molecular interactions among proteins distributed in all cell compartments. Analysis of these interactions has been one of the main topics in cellular and developmental research, and has been mostly achieved by the manipulation of proteins of interest (POIs) at the genetic level. Although genetic strategies have significantly contributed to our current understanding, targeting specific interactions of POIs in a time- and space-controlled manner or analysing the role of POIs in dynamic cellular processes, such as cell migration or cell division, would benefit from more-direct approaches. The recent development of specific protein binders, which can be expressed and function intracellularly, along with advancement in synthetic biology, have contributed to the creation of a new toolbox for direct protein manipulations. Here, we have selected a number of short-tag epitopes for which protein binders from different scaffolds have been generated and showed that single copies of these tags allowed efficient POI binding and manipulation in living cells. Using *Drosophila*, we also find that single short tags can be used for POI manipulation *in vivo*.

## Abstract (precis)

**Summary:** Single copies of short epitope tags were inserted into proteins of interest, allowing for *in vivo* binding and manipulation of the resulting chimeric proteins by genetically encoded epitope binders.

## INTRODUCTION

A key question in cell and developmental biology is how the millions of protein molecules present in any given cell regulate cellular functions in a predictable and coordinated manner. Much of the work carried in the past decades to study protein function in their *in vivo* setting has relied on the use of genetic and reverse genetic approaches that, when combined with biochemical and structural studies, have been extremely successful in gaining insight into protein function (Housden et al., 2017; Wang et al., 2016). However, it emerged that most proteins can interact with many different partners, often in a location- or context-dependent fashion, in many cases regulated by specific post-translational modifications. The complexity of protein-protein interactions has made it very difficult to decipher the manifold properties of any given protein of interest (POI) by using existing gain- and loss-of-function genetic studies. It would be desirable to have at hand a diversified toolbox to manipulate proteins directly in time and space in more controllable fashion.

Over the past few years, several novel approaches have opened up the way to specifically and directly manipulate the function of POIs in different ways in living cells or organisms, and to analyse the consequences of such manipulation at the cellular or organismal level. On the one hand, optogenetic tools have allowed users to manipulate proteins by fusing them to optically regulated modules using light as an inducer. These tools are mostly based on the properties of specific natural occurring photosensitive proteins to change their conformation or aggregation state in response to specific wavelengths (Tischer and Weiner, 2014). These proteins have been engineered into optogenetic systems to control neuronal activity (Rost et al., 2017), direct subcellular localization (Buckley et al., 2016; Niopek et al., 2016), turn protein functionality on or off (Bonger et al., 2014), promote gene expression or repression (Müller et al., 2015), or induce protein degradation and regulate cell signalling (Repina et al., 2017; Zhang and Cui, 2015). Alternatively, chemically regulated modules can also be fused to POIs such that some of their functions (half-life, localization, etc.) can be manipulated (Banaszynski et al., 2006; Bonger et al., 2011; Chung et al., 2015; Czapiński et al., 2017; Natsume and Kanemaki, 2017; Natsume et al., 2016).

On the other hand, protein binders such as scFvs, nanobodies, DARPins, Affibodies, Monobodies and others have been used to directly target and manipulate POIs in different cellular environments (extracellular or different intracellular compartments) (Gebauer and Skerra, 2020; Gilbreth and Koide, 2012; Harmansa and Affolter, 2018; Helma et al., 2015; Holliger and Hudson, 2005; Ingram et al., 2018; Plückthun, 2015; Sha et al., 2017; Škrlec et al., 2015). These protein binders can be functionalized to allow the regulation of POIs in a desired manner. Using functionalized protein binders, POIs can be visualized, degraded, delocalized or post-transcriptionally modified *in vivo* in order to learn more about the function of the POIs in cultured cells or in developing organisms (Aguilar et al., 2019a; Bieli et al., 2016; Harmansa and Affolter, 2018; Prole and Taylor, 2019; Schumacher et al., 2018).

Several strategies allow the targeting and manipulation of POIs *in vivo* via the use of protein binders. Binders against proteins can be isolated using existing platforms and/or libraries, functionalized in a desired manner and expressed in cells or organisms upon transfection, viral transduction or from transgenes inserted into the genome (Dong et al., 2019; Dreier and Plückthun, 2012; Fridy et al., 2014; McMahon et al., 2018; Moutel et al., 2016; Röder et al., 2017; Woods, 2019). Alternatively, binders against fluorescent tags can be used to manipulate a POI that has been fused to a fluorescent protein (FP). This strategy has the advantage that well validated FP binders are available, and that the fusion protein can be visualized during the process using confocal microscopy (Kaiser et al., 2014; Prole and Taylor, 2019). Ideally, and to minimize the potential perturbation of the POI, the latter could be tagged by a short peptide to which high-affinity protein binders have been identified and characterized; this approach would allow the use of available, well-characterized and validated binders, and results in minimal potential disturbance of the function of the POI. Multiple protein manipulation tools generated with nanobodies or DARPins directed towards FPs (Aguilar et al., 2019a; Beghein and Gettemans, 2017; Brauchle et al., 2014; Schumacher et al., 2018; Vigano et al., 2018) could be adapted in order to functionalize small tag binders.

Here, we have selected a number of existing short-tag epitopes for which protein binders from different scaffolds have been reported in the past few years. We have tested whether these tags can be bound by the corresponding protein binders in living cells when they are inserted in a single copy in a POI. We indeed find that, in most cases, a single copy of a short tag allows protein binding and manipulation. Using *Drosophila*, we show that single short tags can also be recognized *in vivo* in developing organisms and allow protein degradation and protein relocalization. Using combinations of these short tags and their corresponding well-characterized binders will allow many interesting protein manipulations with minimal functional interference.

## RESULTS

We wanted to investigate whether small tag binders [such as single chain fragments v (scFv) and nanobodies (Nb)], which were shown to work *in vivo* as intrabodies, were able to bind single short peptide tags inserted in proteins located in different cellular compartments.

**Fig. 1.**
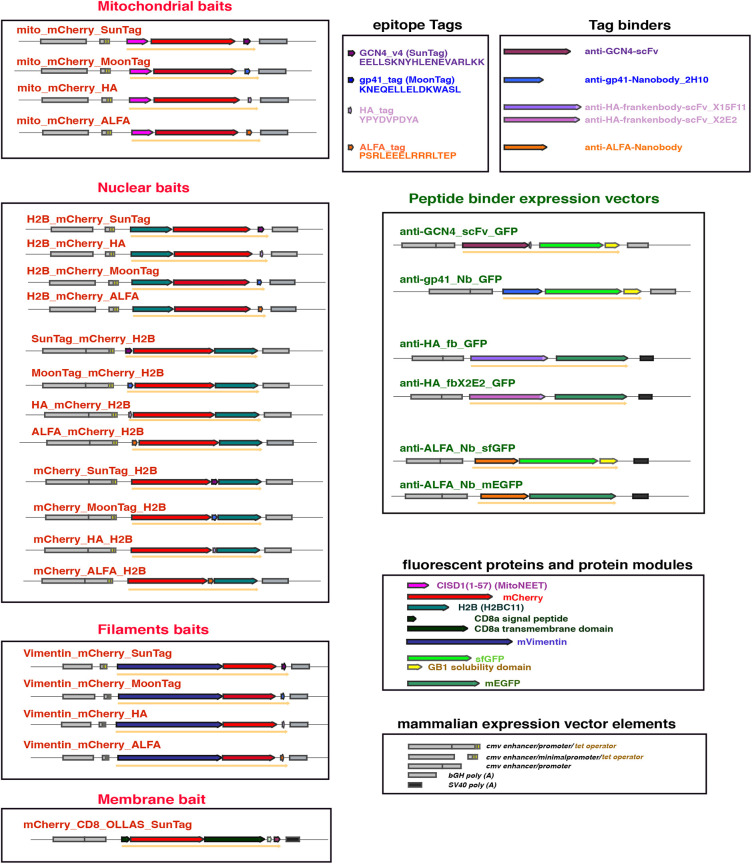
**Schematic representation of the constructs.** The transcriptional elements [enhancer, promoter and poly (A) adenylation] of the different mammalian expression vectors are depicted as grey filled boxes. The different protein-coding modules are represented as coloured block arrows, while the resulting fusion protein is depicted as a solid orange arrow below the modules.

We used transient transfection in HeLa cells as a model system to test the binding properties of these protein binders (Brauchle et al., 2014; Moutel et al., 2016; Vigano et al., 2018). We therefore generated mammalian expression constructs for the anti-GCN4 (SunTag) scFv (Tanenbaum et al., 2014), the anti-gp41(MoonTag) nanobody 2H10 (Boersma et al., 2019), the anti-HA (frankenbodies) scFvs (Zhao et al., 2019) and the anti-ALFA (Götzke et al., 2019) nanobody, each fused to either sfGFP or mEGFP for intracellular visualization. All the binders were expressed under the control of the strong CMV promoter/enhancer (Fig. 1). We next generated differently localized cellular ‘baits' containing one single copy of each tag fused to different proteins or protein domains for localization purposes, and to mCherry for visualization (Fig. 1).

The mitochondrial baits contain the N-terminal domain of the protein MitoNEET (CISD1), which is anchored to the outer membrane of the mitochondria and exposed to the cytoplasmic environment (Colca et al., 2004; Wang et al., 2017). This domain was fused to mCherry and to a single copy of the tags we tested (GCN4-v4, 19 amino acids; gp41, 15 amino acids; HA, 9 amino acids; ALFA, 15 amino acids) in the C-terminal position. The expression patterns of these different mitochondrial constructs in transfected cells were very similar (Fig. S1A), with most of the mitochondria around the nuclei decorated by the mCherry protein and with almost no expression visible in the cytoplasm, but some localized accumulation in additional dots, possibly representing other internal membrane compartments. We also noted a slightly different distribution for the mito_mCherry_MoonTag (Fig. S1Ab): the mitochondria appeared less rounded and more filamentous, and the cytoplasmic mCherry signal was slightly stronger. A stronger cytoplasmic signal was observed for mito_mCherry_ALFA (Fig. S1Ad).

The nuclear baits were based on histone H2B (H2BC11) fused to mCherry either at the N- (H2B_mCherry) or C- (mCherry_H2B) terminus, and with the individual tags located at the N-terminus (Tag_mCherry_H2B), between mCherry and H2B (mCherry_Tag_H2B) or at the C-terminus (H2B_mCherry_Tag). All these nuclear baits were located exclusively to the nucleus upon transient expression, although some appeared more concentrated in nucleoli or unspecific nuclear bodies, irrespective of the position of the H2B or the peptide tags (Fig. S1B). The different localizations in the nucleus might be due to an accumulation in specific sub-nuclear structures for coping with the overexpression (Amer-Sarsour and Ashkenazi, 2019; Rekulapally and Suresh, 2019) or might reflect the different localization of the H2B fusion protein during the cell cycle phases (Duronio and Marzluff, 2017; Kurat et al., 2014; Romeo and Schumperli, 2016). Moreover, it could also reflect the rapid turnover of the histone H2B specifically in chromatin domains with high transcriptional activity (Kimura and Cook, 2001).

We also generated a bait with the leader sequence and the transmembrane domain of the mouse CD8 protein fused to mCherry and containing both the OLLAS (Park et al., 2008) and the GCN4-v4 tags. In *Drosophila melanogaste*r, this construct arrangement has been shown to be inserted into the plasma membrane, exposing the mCherry moiety in the extracellular space and the domains at the C-terminus of CD8 at the cytoplasmic side of the membrane (Harmansa et al., 2017). In the mammalian system, fusion constructs to the CD8 protein domains have been used, for example, to study trans Golgi vesicular transport (Nickel et al., 1998; Pascale et al., 1992a,b). In transfected HeLa cells, mCherry_CD8_OLLAS_SunTag localized to the plasma membrane and to other membranous and filamentous structures inside the cytoplasm (Fig. S1Ai).

The last subcellular bait was a fusion between the mouse vimentin protein, mCherry and one copy of each peptide tag at the C-terminus (Götzke et al., 2019). These constructs reflected the expression of vimentin in the intermediate filaments of the transfected cells (Fig. S1Ae-h), although in the case of the HA tag, the filaments appeared slightly shorter and thicker, occasionally resembling a punctuate structure (Fig. S1Ag).

### SunTag

**Fig. 2.**
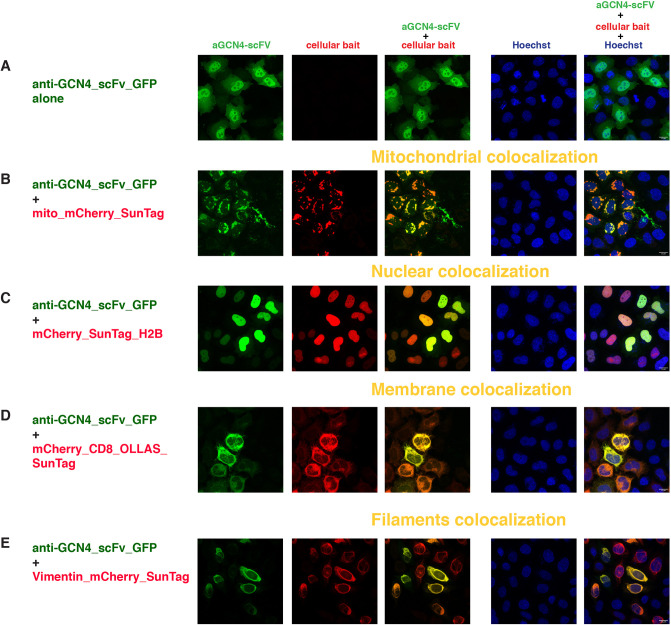
**Intracellular binding of anti-GCN4 scFv (SunTag system).** Confocal images of HeLa cells transiently transfected with (A) anti-GCN4_scFv_GFP alone or with (B-E) the combination of anti-GCN4_scFv_GFP and (B) mito_mCherry_SunTag, (C) mCherry_SunTag_H2B, (D) mCherryCD8_OLLAS_SunTag or (E) vimentin_mCherry_SunTag. The first column represents the GFP channel (green), the second column is the mCherry channel (red), the third column is the overlay of the two channels, showing the colocalization (indicated in yellow) of the anti-GCN4_scFv with the respective mitochondrial (B), nuclear (C), membrane (D) and filament (E) baits; the fourth column represents the nuclear Hoechst staining (blue) and the fifth column is the merge of all three channels. Scale bars: 15 µm. Images were taken 24 h post-transfection. Transfected constructs are indicated at the left of each row and the single and merge channels are indicated at the top of the respective columns. The figures are from a representative experiment, performed at least three times.

The SunTag system was developed to visualize protein expression and translation in high resolution fluorescence imaging (Tanenbaum et al., 2014). The tag (v1) is an epitope derived from the yeast amino acid starvation-responsive transcription factor GCN4, subsequently optimized (v4) for binding to a previously characterized scFv with specific intracellular expression (Wörn et al., 2000). The anti-GCN4_scFv_GFP was uniformly distributed both in the cytoplasm and in the nucleus of the transfected cells, with a stronger green signal in the nucleus (Fig. 2A, Fig. S2A). This nuclear signal was not entirely overlapping with the Hoechst staining (which highlights mostly the DNA), indicating free diffusion of the scFv in the nucleoplasm. Occasionally, we observed some aggregation/accumulation in some unidentified granular structures in the cytoplasm, possibly owing to the high level of expression of the construct.

Co-expression of anti-GCN4_scFv_GFP with the mitochondrial bait carrying a single copy of the GCN4 epitope v4 significantly changed the distribution of the anti-GCN4_scFv_GFP, relocalizing it to the outer mitochondrial membrane (Fig. 2B). Mitochondrial localization of mito_mCherry_SunTag was not altered by co-expression of anti-GCN4_scFv_GFP (compare Fig. S1Aa with Fig. 2B).

It has to be noted that not all the anti-GCN4_scFv_GFP molecules were recruited to the mitochondria, as seen by residual GFP signal in the cytoplasm, presumably owing to the limited number of CISD1 binding partners at the mitochondrial surface. Varying the ratio of the transfected DNAs did not change the amount of anti-GCN4_scFv_GFP observed at the mitochondria (data not shown).

Importantly, mitochondrial recruitment was specific, as we did not observe any colocalization of anti-GCN4_scFv_GFP with similar mitochondrial baits carrying one copy of either the unrelated HA tag (Fig. S3A) or the gp41 (MoonTag) (Fig. S3B). We also generated a mitochondrial bait containing one copy of the original GCN4 peptide tag v1 (Tanenbaum et al., 2014) and observed the same recruitment to the outer mitochondrial membrane of the anti-GCN4_scFv_GFP (data not shown).

We next tested for nuclear colocalization with three different nuclear baits, all based on the histone protein H2B with a single SunTag epitope in different positions (Fig. 1). Co-transfection of these nuclear baits with the anti-GCN4_scFv_GFP (Fig. 2C, Fig. S2B,C) clearly showed nuclear accumulation of the scFv with a nearly complete overlap of the mCherry and GFP signals in the nuclei of transfected cells and barely detectable GFP signal in the cytoplasm. Nuclear recruitment was equally efficient for all the SunTag epitope positions tested.

After co-transfection of the anti-GCN4_scFv_GFP with nuclear baits carrying the HA tag, the MoonTag, the ALFA tag or no tag (mCherry_H2B) (Fig. S3C-H), we observed a partial overlap of the GFP and mCherry signals, especially in the nuclear bodies, but the majority of the anti-GCN4_scFv_GFP was still visible in the cytoplasm and in the nucleoplasm, with a cellular localization very similar to that observed in the absence of any bait. The strongest overlap was observed with ALFA tag, possibly owing to a certain similarity of the two tags (see Discussion).

We then tested the binding and localization of the anti-GCN4_scFv_GFP in the presence of the membrane bait. As mentioned above, mCherry_CD8_OLLAS_SunTag localized both at the plasma membrane and at other filamentous structures associated with internal membranes of the transfected cells (Fig. S1Ai). Its localization did not change when co-transfected with the anti-GCN4_scFv_GFP, but it was able to bind and recruit the scFv, as illustrated by the almost complete overlap of the GFP and mCherry signal (Fig. 2D). Finally, when co-transfected with the vimentin_mCherry_SunTag bait, we observed an almost complete relocalization of the anti-GCN4_scFv_GFP to the intermediate filaments (Fig. 2E), supporting an efficient *in vivo* binding of the anti-GCN4_scFv_GFP to yet another subcellular compartment exposing a single copy of the SunTag. We also confirmed no binding of the anti-GCN4_scFv_GFP to a vimentin bait with the MoonTag (Fig. S3I) and some cross-reactivity with the vimentin bait containing the ALFA tag (Fig. S3J).

### MoonTag

The MoonTag system (Boersma et al., 2019) is based on the epitope from the membrane-proximal external region of the human HIV-1 envelope glycoprotein subunit gp41 and its nanobody binder 2H10. We cloned the nanobody anti-gp41 2H10 fused to sfGFP_GB1, in a CMV promoter/enhancer expression vector (anti-gp41_Nb_GFP) and tested its localization with the different cellular baits containing the gp41 epitope (MoonTag) (Fig. 1).

**Fig. 3.**
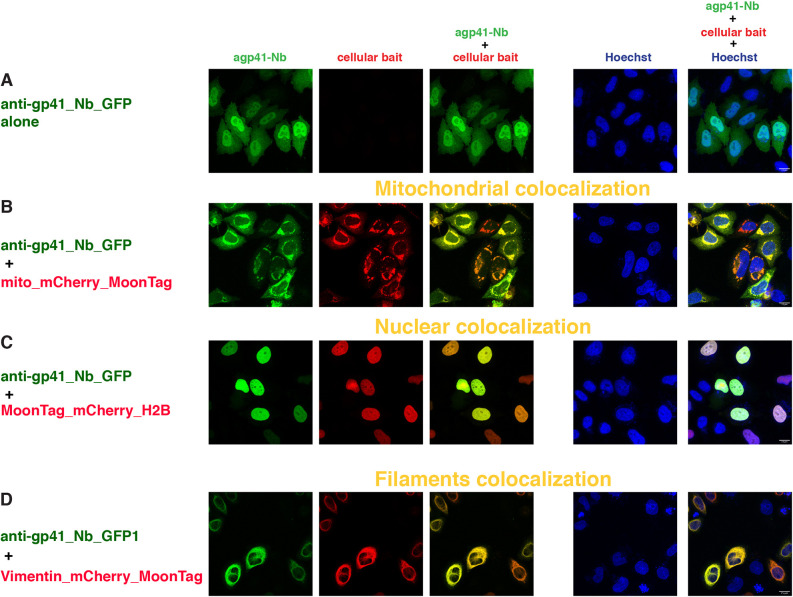
**Intracellular binding of anti-gp41 nanobody (MoonTag system).** Confocal images of HeLa cells 24 h after transient transfection with constructs indicated at the left of each row and imaged for the channels indicated at the top of each row. The first column represents the GFP channel (green), the second column is the mCherry channel (red), the third column is the overlay of the two channels, showing the colocalization (indicated in yellow) of the anti-gp41_Nb_GFP with the respective mitochondrial (B), nuclear (C), and filament (D) baits; the fourth column represents the nuclear Hoechst staining (blue) and the fifth column is the merge of all three channels. Scale bars: 15 µm. The figures are from a representative experiment, performed at least three times.

Expression of the anti-gp41_Nb_GFP alone resulted in rather uniform distribution of the protein in the cytoplasm (Fig. 3A, Fig. S2D) and, as observed for the anti-GCN4_scFv_GFP, a stronger signal in the nuclei. We never observed any aggregation, possibly reflecting the better solubility of the nanobody than the scFv and confirming its good intracellular expression. In the nuclear colocalization assay, the anti-gp41_Nb_GFP was very efficiently recruited to the nuclei by all three nuclear baits (Fig. 3C, Fig. S2E,F); furthermore, no GFP signal was detected in the cytoplasm.

When the anti-gp41_Nb_GFP was co-transfected with the nuclear bait carrying no tag (mCherry_H2B) or H2B_mCherry_SunTag (Fig. S4E,F), we also observed some overlapping GFP signal in the nuclear bodies with strong accumulation of the mCherry signal, but the majority of the GFP signal was uniformly distributed within the nucleus and the cytoplasm, where no mCherry signal was detected. As observed with the anti-GCN4_scFv_GFP in the similar combination set up, these results are indicative of no binding or active recruitment by the nuclear baits with a different tag or with no tag.

In co-transfection experiments with the anti-gp41_Nb_GFP and the mitochondrial bait carrying one copy of the MoonTag (Fig. 3B), we observed redistribution of the anti-gp41_Nb_GFP to the outer mitochondrial membrane, although there was some detectable GFP signal in the cytoplasm and the nucleus.

Co-transfection of the anti-gp41_Nb_GFP with mitochondrial baits either containing the HA, the SunTag or the ALFA tag (Fig. S4A-C) also showed a very partial overlap of the GFP and the mCherry signals, mostly with HA; however, most of the GFP signal remained uniformly distributed in the cytoplasm and the nucleus (especially with the mito_mCherry_SunTag), with no indication of binding or active recruitment.

The colocalization of the anti-gp41_Nb_GFP to the intermediate filaments was also very prominent (Fig. 3D), indicating a very efficient binding and recruitment to these structures by vimentin carrying one copy of the MoonTag. Furthermore, we did not observe any cross-reactivity with vimentin carrying the SunTag or the ALFA tag (Fig. S4G,H).

### HA tag

The HA peptide derived from the influenza virus hemagglutinin has been extensively used in biochemical studies due to the availability of high-affinity monoclonal antibodies (Field et al., 1988; Wilson et al., 1984). Recently, two different anti-HA scFvs derived from the monoclonal anti-HA antibody 12CA5 were generated and called frankenbodies (Zhao et al., 2019). The two frankenbodies anti-HA-scFvX15F11 and anti-HA-scFvX2E2 were made by grafting the complementarity determining regions (CDRs) of the 12CA5 monoclonal antibody into two different scFv scaffolds with a demonstrated solubility *in vivo*. We tested the function of these two anti-HA_scFvs as intrabodies for their binding to a single copy of the HA epitope embedded in the same cellular baits as developed analogously for the SunTag and MoonTag systems (Fig. 1).

**Fig. 4.**
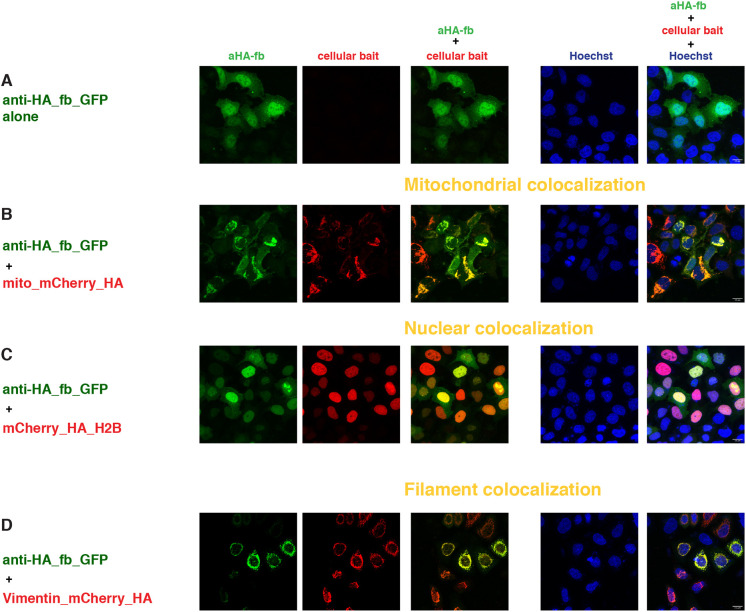
**Intracellular binding of anti-HA_fb_GFP (HA system).** Confocal images of HeLa cells 24 h after transient transfection with constructs indicated at the left of each row and imaged for the channels indicated at the top of each row. The first column represents the GFP channel (green), the second column is the mCherry channel (red), the third column is the overlay of the two channels, showing the colocalization (indicated in yellow) of the anti-HA_fb_GFP with the respective mitochondrial (B), nuclear (C), and filament (D) baits; the fourth column represents the nuclear Hoechst staining (blue) and the fifth column is the merge of all three channels. Scale bars: 15 µm. The figures are from a representative experiment, performed at least three times.

The expression pattern of the two frankenbodies in the single transfection conditions in the absence of any bait was uniform in both nucleus and cytoplasm, with stronger GFP signal in the nucleoplasm (Fig. 4A, Fig. S5A), confirming observations made in a different cell line (U2OS) (Zhao et al., 2019).

Co-transfection with the mitochondrial bait containing one copy of the HA epitope (mito_mCherry_HA) showed significant recruitment to the outer mitochondrial membrane of both frankenbodies (Fig. 4B, Fig. S5B). Although the assay is not quantitative, the fraction of scFvs, which was detected in the cytoplasm or nucleoplasm seemed higher for anti-HA_fb_GFP than for anti-HA_fbX2E2_GFP. The residual GFP signal not localizing at the mitochondrial membrane was also higher for these anti-HA_scFvs than the anti-GCN4_scFv signal in equivalent conditions (Fig. 2B). This may reflect a lower binding affinity of the scFvs to the HA epitope and consequently a lower efficiency of recruitment with a single epitope copy, and would be in agreement with the lower signal-to-noise ratio of the Mito_mCherry_1×HA versus Mito_mCherry_smHA, containing 10×HA, reported by Zhao et al. (2019). We also observed a slight colocalization of the frankenbodies with mitochondrial baits containing the other tags, with overlapping GFP and mCherry signals of different intensity and patterns in each combination (Fig. S6A-D).

We next tested whether the nuclear baits containing one copy of the HA epitope positioned in different locations of the proteins were able to bind and recruit the frankenbodies to chromatin. There was a clear nuclear colocalization under all the conditions tested, with a higher efficiency for the anti-HA_fbX2E2_GFP than for the anti-HA_fb_GFP, as judged from the residual GFP signal in the cytoplasm (Fig. 4C, Fig. S5A′,B′, Fig. S5C-E).

Transfection of anti-HA_scFvs with the mCherry_H2B resulted in some overlapping GFP and mCherry signals in the nucleoli/nuclear bodies (as seen with the other protein binders), but the majority of the GFP signal was in the cytoplasm/nucleoplasm of transfected cells (Fig. S7A,B). In co-transfection experiments with nuclear baits containing the ALFA tag (Fig. S7C,D), we also observed minimal overlap of the mCherry and mEGFP signals in the nuclei. Hence, a single copy of the HA epitope, regardless of the insertion position, appeared sufficient to specifically bind and recruit the frankenbodies to the nucleus, although somewhat less efficiently than the MoonTag or SunTag counterparts. We did not observe an overlap of anti-HA_fb_GFP with the unrelated bait mCherry_CD8_OLLAS_SunTag (Fig. S6E).

Co-transfection experiments of the two frankenbodies with vimentin_mCherry_HA confirmed the binding to a single copy of the epitope in cultured cells, although we observed a higher residual GFP signal both in the cytoplasm and the nucleoplasm (Fig. 4D, Fig. S5F). Furthermore, as mentioned earlier, the expression of the vimentin_mCherry_HA, either alone (Fig. S1Ah) or with the anti-HA_scFvs, was significantly different from the intermediate filaments painted by the vimentin_mCherry-SunTag or MoonTag, indicating a possible disruption of the filament structure. Nevertheless, the two anti-HA_scFvs were able to bind to this HA bait specifically as they did not show binding to vimentin_mCherry_ALFA (Fig. S7E,F).

### ALFA tag

**Fig. 5.**
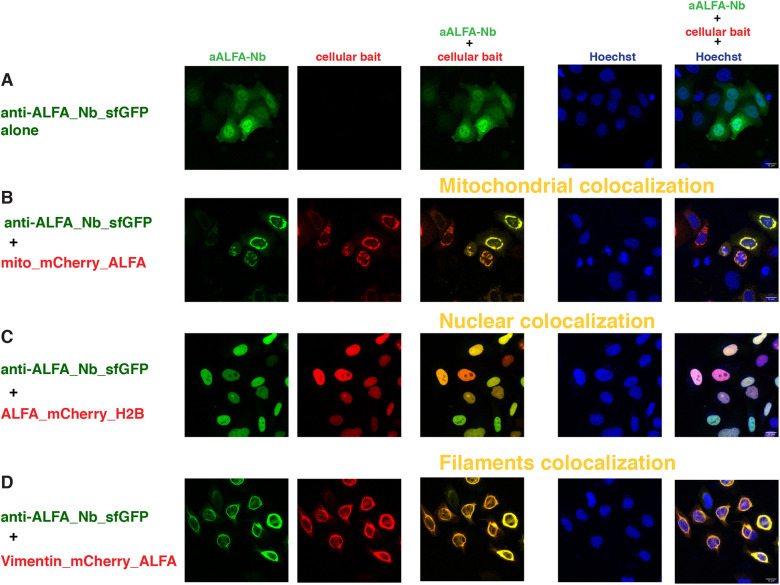
**Intracellular binding of anti-ALFA_Nb_sfGFP (ALFA tag system).** Confocal images of HeLa cells 24 h after transient transfection with constructs indicated at the left of each row and imaged for the channels indicated at the top of each row. The first column represents the GFP channel (green), the second column is the mCherry channel (red), the third column is the overlay of the two channels, showing the colocalization (indicated in yellow) of the anti-ALFA_Nb_sfGFP with the respective mitochondrial (B), nuclear (C) and filament (D) baits; the fourth column represents the nuclear Hoechst staining (blue) and the fifth column is the merge of all three channels. Scale bars: 15 µm. The figures are from a representative experiment, performed at least three times.

Recently, Götzke et al. developed the ALFA tag system, which is based on a short synthetic tag and its nanobody binder (Götzke et al., 2019). We decided to test this new system as well, as we reasoned that nanobodies might be somewhat more versatile than scFvs as protein binders *in vivo* (see Discussion). Therefore, we generated an anti-ALFA nanobody construct fused either to sfGFP_GB1 or mEGFP and confirmed that the fusion proteins were well expressed in transfected cells (Fig. 5A, Figs S8A and S9Aa,b), as reported by Götzke (2019). In the case of anti-ALFA nanobody fused to mEGFP, we occasionally observed some minor aggregation (data not shown), but the overall distribution pattern of anti-ALFA nanobodies fused to sfGFP or mEGFP was very similar to the binders tested above.

In co-transfection experiments with the mito_mCherry_ALFA bait, the binding and recruitment to the outer mitochondrial membrane of the anti-ALFA nanobody was very efficient (Fig. 5B, Fig. S8B), while residual cytoplasmic signal was virtually negligible. Control experiments with mitochondrial baits containing the MoonTag (Fig. S9Ba,b) revealed no cross-reactivity.

The nuclear colocalization was also very efficient with all the nuclear baits tested, irrespective of the position of the ALFA tag (Fig. 5C, Fig. S8A′,B′,C-E). Control experiments with nuclear baits containing different tags showed a detectable nuclear colocalization with the H2B_mCherry_SunTag and a partial overlap with the mCherry_H2B signal, although most of the signals of the nanobodies were still detectable in the cytoplasm (Fig. S9Ac-h).

Finally, we tested binding and recruitment to the intermediate filaments with the vimentin_mCherry bait carrying one copy of the ALFA tag at the C-terminus. As reported with a similar vimentin construct, but with the ALFA tag at the N-terminus (and without FP) (Götzke et al., 2019), we observed excellent colocalization of the mCherry and GFP signals (Fig. 5D, Fig. S8F). Furthermore, we did not observe any cross-reactivity with vimentin-SunTag or MoonTag (Fig. S9Bc-f).

### Binding and manipulation of single HA-tagged proteins *in vivo*

**Fig. 6.**
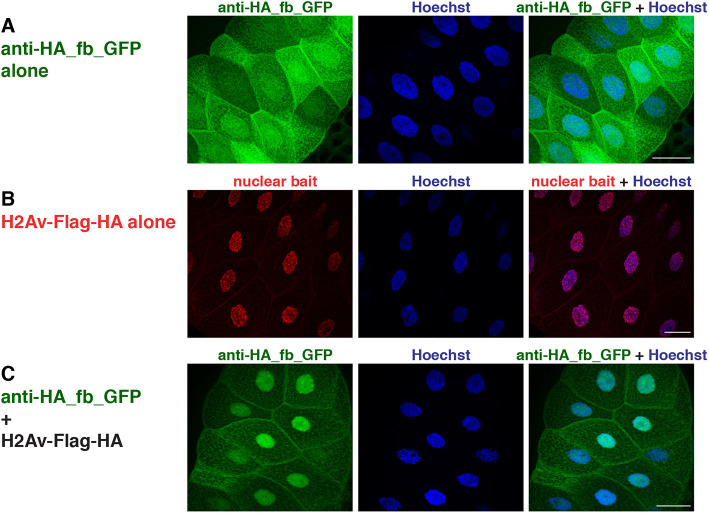
**Intracellular binding of anti-HA_fb_GFP (HA system) *in vivo*.** (A-C) Confocal images of salivary glands from 3rd instar *Drosophila* larvae expressing the UAS constructs indicated at the left of each row using a brk-GAL4 driver. Single and merge channels are indicated at the top of the respective panel. Nuclei are visualized by Hoechst staining (blue). Scale bars: 50 µm.

We next addressed whether single tagged POIs can be recognized and manipulated by the respective binders *in vivo*. We used *Drosophila* as a test system and focused on the HA tag, as this epitope is widely used to mark proteins in the *Drosophila* research field. We generated transgenic flies expressing an anti-HA_fb_GFP fusion protein under the control of the UAS/GAL4 system. When expression was activated in salivary glands, the GFP signal was distributed throughout the cell; similarly to the co-transfection experiments, GFP levels were slightly increased in the nuclei (Fig. 6A). Co-expression of nuclear-localized histone H2Av carrying a single HA tag at the C-terminus (H2Av-Flag-HA) resulted in a strong accumulation of the anti-HA_fb_GFP in the nucleus (Fig. 6B,C). Similar to what we observed in the corresponding cell culture experiment (Fig. 4 and Fig. S5), the cytoplasmic pool of anti-HA_fb_GFP was reduced but not completely depleted. To address whether the efficacy of nuclear translocation might depend on the number of epitope tag copies, we used *Drosophila* Histone H4 carrying three HA copies at its C-terminus as nuclear bait (H4-3×HA). Indeed, using the same experimental setting, co-expression of anti-HA_fb_GFP with H4-3×HA (Fig. S10) resulted in strong accumulation of the GFP signal in the nucleus and its complete depletion from the cytosol. Cumulatively, these findings suggest that HA binders can be used for efficient binding of proteins *in vivo*, with the efficiency being somewhat influenced by the copy number of HA epitopes.

**Fig. 7.**
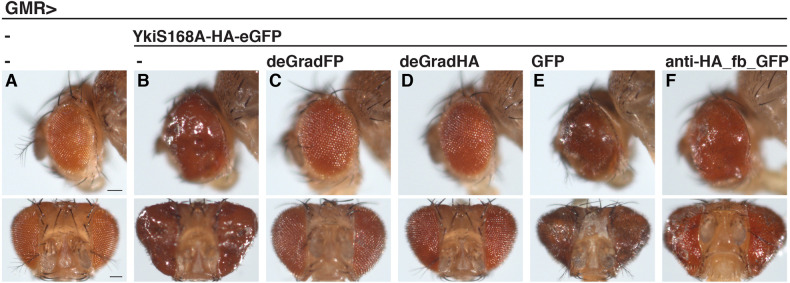
**Manipulation of HA-tagged proteins by deGradHA *in vivo*.** (A-F) Side (top row) or frontal (bottom row) views of *Drosophila* adult eyes carrying the eye-specific GMR-GAL4 driver alone (A), or in combination with the UAS constructs indicated at the top (B-F). Scale bars: 100 µm.

We also tested whether single-tagged POIs can be inactivated by functionalized protein binders. Previous work established a tool, deGradFP, allowing for ubiquitin/proteasome degradation of GFP-tagged proteins using a nanobody against GFP (Caussinus et al., 2011). In this system, a single-domain antibody fragment against GFP (vhhGFP4) is replacing the substrate specificity domain of the *Drosophila* E3 ligase component Slmb, thereby generating a complex that is directed against GFP and GFP-tagged proteins. We modified the deGradFP tool by replacing the vhhGFP4 domain with the anti-HA-frankenbody-scFvX15F11 to generate deGradHA, and tested its activity towards HA-tagged proteins in transgenic flies. First, we turned to Yorki (Yki, *Drosophila* YAP/TAZ), a transcriptional co-activator that is regulated through phosphorylation by the Hippo signalling pathway to control cell proliferation and organ size (Huang et al., 2005). In the construct we used, YkiS168A-HA-eGFP, Yki contains a point mutation that renders the protein hyperactive in promoting organ growth (Oh and Irvine, 2008). In addition, the protein contains a C-terminal single HA tag followed by GFP. As shown before (Oh and Irvine, 2008), transgenic flies expressing YkiS168A-HA-eGFP using an eye-specific driver displayed massive tissue overgrowth (Fig. 7A,B). This phenotype was completely reversed by co-expressing deGradFP or deGradHA but not by co-expression of an unrelated protein (GFP) (Fig. 7C-E), the last of which excludes titration effects of the UAS/GAL4 system. In addition, phenotypic suppression was not visible with anti-HA_fb_GFP, demonstrating that binding alone is not sufficient for the observed effect (Fig. 7F). Thus, the deGradHA tool can efficiently inactivate proteins carrying single HA epitope tags.

**Fig. 8.**
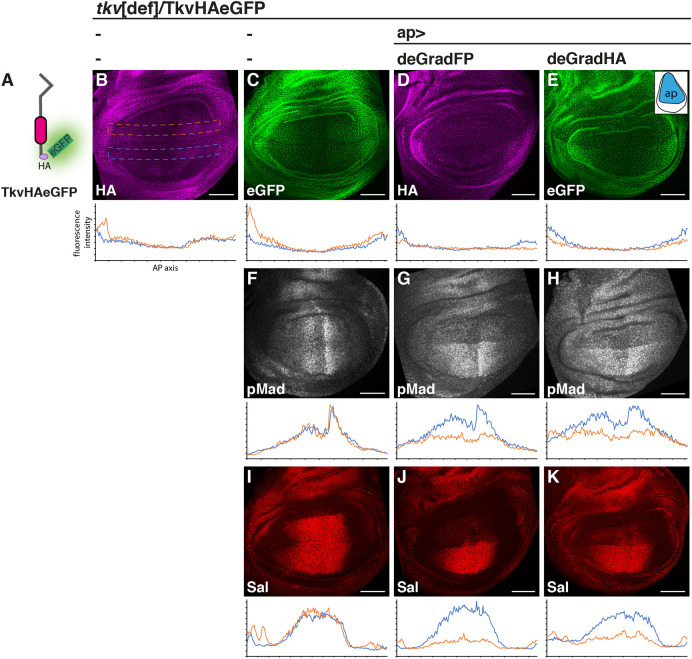
**Manipulation of endogenously HA-tagged proteins by deGradHA.** (A-K) Distribution of TkvHAeGFP (schematically depicted in A) visualized by immunostaining with a HA antibody (B,D) or GFP-autofluorescence (C,E), and pMad (F-H) and Sal (I-K) immunostaining in 3rd instar *Drosophila* wing imaginal discs of the indicated genotypes. The expression domain of the ap-GAL4 driver is schematically shown in the inset in E. Plots below each panel depict relative fluorescent intensity of ventral (control, blue) and dorsal (experimental, orange) cells along the AP axis of the wing pouch (dashed lines in B indicate areas used for quantification). Owing to the low expression of Tkv in the medial pouch, effects of the deGrad tools are better visible in lateral regions. All larvae carry the engineered TkvHAeGFP allele over a chromosomal deletion of the *tkv* locus. Scale bars: 50 µm.

Last, we addressed whether our tools can affect the stability and activity of endogenously tagged POIs *in vivo*. As a POI, we chose the *Drosophila* BMP (Bone Morphogenetic Protein) receptor Thickveins (Tkv), a protein transmitting BMP signals to the nucleus through the direct phosphorylation of the transcription factor Mad. Tkv is essential for most of the *Drosophila* BMP responses, including cases where BMPs act as morphogens such as the larval wing precursor (Hamaratoglu et al., 2014). In this tissue, Tkv is activated by the BMP ligands Dpp and Gbb to generate a gradient of phosphorylated Mad (pMad) along the anterior-posterior (AP) axis of the developing organ. We used genome engineering to introduce sequences coding for a single HA tag followed by eGFP in the *tkv* gene, resulting in a C-terminally tagged receptor (TkvHAeGFP; Fig. 8A). We chose to include single copies of two different epitopes (HA and eGFP) to enable the independent manipulation and visualization of the protein. Flies carrying the tagged allele in homozygosity developed normally and did not display any visible morphological abnormalities. Wing imaginal discs with TkvHAeGFP as the sole Tkv source displayed a normal pMad gradient and develop into phenotypically wild-type wings (Fig. S11). Both epitopes captured the characteristic distribution of Tkv in the 3rd instar wing imaginal disc (Fig. 8B,C). Expression of either deGradFP or the newly established deGradHA (Fig. 8D,E) in the dorsal compartment of the discs using apterous-GAL4 (ap-GAL4) resulted in a clear reduction of Tkv levels in dorsal cells. This reduction in receptor levels was accompanied by a substantial loss of pMad and Spalt (Sal) expression – a BMP/pMad-target gene (de Celis and Barrio, 2009) in the same compartment (Fig. 8F-K). The deGradFP tool appeared slightly more effective than deGradHA, although neither tool fully eliminated Tkv levels and pMad or Sal expression in dorsal cells. To address whether the efficacy of deGradHA could be improved by increasing the HA copy numbers in the POI, we used, in the same assay, a previously described version of Tkv that carries three copies of the HA tag at the C-terminus [Tkv3×HA (Norman et al., 2016)]. Although levels of Tkv cannot be monitored with this construct due to the absence of a second epitope in the construct, the stronger reduction of dorsal pMad and the nearly complete loss of Sal signal (Fig. S12) suggested that increasing the number of the HA tags improved the performance of deGradHA.

## DISCUSSION

### Single copies of short peptides and their binders

We focused our study on short peptide tags for which specific high-affinity binders that are soluble and functional in the intracellular milieu have been characterized. Therefore, we selected the following systems: SunTag (Tanenbaum et al., 2014), MoonTag (Boersma et al., 2019), HA (Zhao et al., 2019) and ALFA (Götzke et al., 2019). For other commonly used tags such as FLAG (Hopp et al., 1988) or Myc (Evan et al., 1985), we are not aware of specific binders derived from the corresponding monoclonal antibodies that perform as intrabodies (Fujiwara et al., 2002; Marschall et al., 2015; Moutel et al., 2016; Wörn et al., 2000).

Recently, a number of other short peptide binders were characterized, such as the BC2 nanobody recognizing the N-terminal amino acids 16-27 of β-catenin (Traenkle et al., 2015), the KTM219-derived scFv binding to a stretch of seven amino acids of the BGPC7 (bone Gla protein or osteocalcin) (Wongso et al., 2017) and the nanobody NbSyn2 recognizing the C-terminal of α-Synuclein [EPEA C-tag (De Genst et al., 2010)]. Although they were shown to work intracellularly as chromobody or flashbody, we did not investigate them, as they recognize and bind to the corresponding endogenous proteins. Another binder, the nanobody VHH05 binding to a 14 amino acid peptide epitope of the E2 ubiquitin-conjugating enzyme UBC6e (Ling et al., 2019), was published after we had initiated our studies.

We were particularly interested in testing whether the binders would be able to efficiently bind to a single copy of the selected tag *in vivo*. If this were the case, proteins of interest could be minimally modified with the aim of not affecting any of their *in vivo* functions. Furthermore, current technology of precise gene knock-in or tagging might be more efficient with short insertions in some organisms, such as zebrafish.

We did observe that the various tags, even in single copy, mildly altered the expression of certain POIs examined. The insertion of ALFA and MoonTag into the mitochondrial bait (mito_mCherry_MoonTg and mito_mCherry_ALFA) slightly altered the mitochondrial ‘shape' and resulted in residual cytoplasmic signal upon overexpression (Fig. S1A). Götzke et al. (2019) used a slightly different mitochondrial bait with one copy of ALFA tag and they did not report a similar pattern of expression; moreover, the same mitochondrial bait with 12 copies of the MoonTag was tested in another cellular context (Boersma et al., 2019). The insertion of the HA tag into vimentin (vimentin_mCherry HA) also slightly altered the appearance of the filaments painted by the mCherry signal (Fig. S1a). We think that it might be more likely a consequence of overexpression rather than a direct influence of the specific tags [or mCherry-tag(s) module]. With the exception of the ALFA system and the anti-HA_frankenbody, the SunTag and MoonTag systems were previously tested *in vivo* in a similar setup to ours, but with cellular baits containing multiple copies of the corresponding tag, to visualize *in vivo* translation at a single molecule resolution (Boersma et al., 2019; Tanenbaum et al., 2014).

### Expression of the binders

We confirmed that all the tested small tag binders, the scFvs (anti-GCN4 and anti-HA frankenbodies) and the nanobodies (anti-gp41 and anti-ALFA) were excellent intrabodies and chromobodies; they were expressed at high levels inside the cells and diffused freely both in the cytoplasm and in the nucleoplasm. We occasionally observed some minor aggregation with the anti-GCN4_scFv_GFP, probably owing to the high overexpression from a CMV promoter, and with both anti-HA frankenbodies. Moreover, the nanobodies binding MoonTag and ALFA hardly displayed any aggregation when expressed at high level, confirming the high solubility of these protein binders (Beghein and Gettemans, 2017; Ingram et al., 2018; Schumacher et al., 2018).

The choice of FP chosen for the generation of chromobodies (Kaiser et al., 2014; Keller et al., 2019; Moutel et al., 2018) may partially influence its expression and/or function. We noticed that for the anti-ALFA_Nb, which was originally tested with mScarlet (Götzke et al., 2019), fusion to sfGFP was preferable, since we observed a weak interference of the mEGFP over the mCherry signal of some baits. For example, vimentin_mCherry_ALFA had a lower intensity signal when bound to the anti-ALFA_Nb_mEGFP than to the anti-ALFA_Nb_sfGFP. Similarly, the signal intensity of vimentin_mCherry_HA was also lower when bound to either mEGFP-frankenbodies, than when expressed alone. However, fusion to mEGFP resulted in higher and brighter binder signals, especially in the nuclei. Overall, we showed that all the binders tested were able to recognize and bind *in vivo* a single copy of the respective peptide tag embedded in proteins of different cell compartments, albeit with different efficiency and affinity.

The systems based on nanobodies (MoonTag and ALFA) might be more suitable for experiments in nuclear and subnuclear compartment, given their general higher solubility inside the cell. We did not notice significant differences of the SunTag, MoonTag or ALFA for recruiting the respective binders to the mitochondria, to membranes or to filaments. The single HA tag, in our cellular experiments, was sufficient to bind and recruit the corresponding frankenbodies to all the structures analysed, but displayed a lower affinity than the three other tags, in agreement with the reported lower signal-to-noise ratio of the Mito_mCherry_1×HA versus Mito_mCherry_smHA in cells, or of 10×HA-H2B-mCherry versus 1× or 4×HA in zebrafish (Zhao et al., 2019). The lower binding affinity might also correlate with the size of the epitope, as the HA tag is the smallest (nine amino acids). Furthermore, our experiments in *Drosophila* confirmed the positive correlation of the HA copy number and *in vivo* binding. Although this could represent a drawback of the HA system, it might also provide an opportunity for titrating the effects of functionalized HA binders by adjusting the number of the HA copies fused to the POI.

### Combination of multiple tags

Combinatorial tagging of POIs would expand the repertoire of protein manipulation. A possibility would be, for example, to use one tag for visualization and the other tag for specific manipulation, as we demonstrated with TkvHAeGFP *in vivo* (see Aguilar et al., 2019b for a discussion on the use of different tags in the same gene). As pointed out in a recent review, expression levels of the protein binder for visualization of a POI must be carefully controlled for a correct interpretation of the results (Aguilar et al., 2019b). Strategies such as inducibility (Panza et al., 2015), self-transcriptional autoregulating domain fusion (Son et al., 2016) or intrinsic self-stability (Tang et al., 2016), which were developed for nanobodies (Panza et al., 2015; Tang et al., 2016) and fibronectin-derived intrabodies (Son et al., 2016), could be applied to all the small tag binders described here.

Our control experiments using baits containing tags that were not supposed to be recognized by the different binders revealed some cross-reactivity between the SunTag and the ALFA systems, mostly with the anti-GCN4_scFv_GFP recognizing the ALFA tag rather than the reverse (Fig. S3G,J). The similarity of the two tags is restricted to three amino acids (EEL), but this might be sufficient for low-affinity binding in that particular context. No other significant cross-reactivity was observed among the other systems, confirming the suitable orthogonality described for SunTag and MoonTag by Boersma et al. (2019). Any combination of two or even three tags would certainly be beneficial for some experiments, with the avoidance of SunTag/ALFA pair.

### Functionalization of small tag binders

We demonstrate the ability of the binders to be recruited by single-tagged anchored proteins to different cellular compartments. The reverse approach, i.e. move or trap the single-tagged POI with an anchored binder, is a possible functionalization of these small tag binders. Mislocalization or trapping of some POIs, tagged with FP, has been developed with anti-GFP nanobodies (Harmansa et al., 2017; Seller et al., 2019), anti-mTFP DARPin (Vigano et al., 2018) and anti-mCherry nanobody (Prole and Taylor, 2019), but also with nanobodies against endogenous, non-tagged proteins, e.g. gelsolin and CapG (Van Audenhove et al., 2013). Moreover, a very recent report showed the efficient trapping of an extracellular POI (Dpp), endogenously tagged with a single HA copy, using a functionalized anti-HA_scFv (Matsuda et al., 2020 preprint).

Another possibility is the addition of ‘degrons' to the small tag binders (Natsume and Kanemaki, 2017), to achieve specific and temporally controlled degradation of the tagged POIs. This approach has been successfully applied using the anti-FP nanobodies (Aguilar et al., 2019a; Beghein and Gettemans, 2017; Deng et al., 2020; Ingram et al., 2018; Prole and Taylor, 2019). Here, we expand these findings by demonstrating that a single short HA tag can be used to inactivate POIs using deGradHA, a tool designed to channel HA-tagged POIs to ubiquitin/proteasome dependent degradation. Thus, in addition to relocalization, small tags in single copies can be used to target POIs for proteolysis enabling a spectrum of additional applications. Our finding that the deGradHA tool can be used to inactivate endogenously tagged proteins is particularly interesting given the emergence of large collections of tagged proteins and the constant development of technologies allowing for fast and efficient endogenous tagging in *Drosophila* and other systems (Bischof et al., 2013; Kanca et al., 2017; Nagarkar-Jaiswal et al., 2015; Sarov et al., 2016, 2012).

Finally, addition of any enzymatic domain to the small tag binders would allow the specific modification of the tagged POI, as it was elegantly shown with a minimal Rho kinase domain fused to the GFP nanobody to phosphorylate a GFP-tagged protein in *Drosophila melanogaster* (Roubinet et al., 2017) or with a proximity-directed O-GlcNAcetylation linking the O-GlcNAc transferase activity to the GFP or EPEA nanobody in cell culture (Ramirez et al., 2020). Several recent reviews have highlighted the versatility of the nanobodies for numerous applications both in clinical and biological research (Beghein and Gettemans, 2017; Cheloha et al., 2020; Ingram et al., 2018; Muyldermans, 2020; Schumacher et al., 2018; Yang and Shah, 2020). The various functionalization strategies can be extended to these small tag binders.

An important aspect in developing tools and strategies for acute protein manipulation in cultured cells and in living organisms is the temporal and spatial inducibility and/or reversibility of the manipulation itself. Recent publications have demonstrated the possibility of directly modifying specific nanobodies in order to control their binding to the target protein either with light (Gil et al., 2020; Yu et al., 2019) or with small molecules (Farrants et al., 2020). It will be exciting to extend these types of modification to the small tag binders used in this study in order to achieve this extra level of regulation and expand the toolbox to acutely and reversibly manipulate proteins *in vivo*.

## MATERIALS AND METHODS

### Plasmid construction

All the eukaryotic expression plasmids were generated by specific PCR amplification and standard restriction cloning. Briefly, the mitochondrial baits containing an N-terminal anchor sequence from the human CISD1 protein (the first 59 amino acids) fused to the N-terminus of mCherry, were generated from pcDNA4TO-mito-mCherry-10×GCN4_v4 (Addgene #60914; Tanenbaum et al., 2014) by substituting the 10×GCN4_v4 with each individual tag, PCR amplified with specific primers and inserted with RsrII/SacII sites. The pH2B_mCherry_Tag plasmids were generated from the respective mito_mCherry_Tag, substituting the CISD1 protein with the human H2BC11 (Histone H2B) by restriction cloning. The other nuclear baits Tag_mCherry_H2b and pmCherry_Tag_H2B were also generated by inserting each PCR amplified Tag into mCherry_H2B (a kind gift from E. Nigg's group, Biozentrum, University of Basel, Switzerland). Substitution of CISD1 with PCR amplified mouse vimentin inserted at EcoRI/BamHI sites of each mito_mCherry_Tag generated the filaments baits. mCherry_CD8_OLLAS_SunTag was synthetized at TWIST Bioscience.

The anti-GCN4_scFv_GFP was generated from pHR-scFv-GCN4-sfGFP-GB1-dWPRE (Addgene #60907; Tanenbaum et al., 2014), cut with EcoRI/XbaI and inserted into pcDNA3. The anti-gp41_Nb_GFP was generated from pHR-Nb 2H10 gp41-sfGFP-GB1-dWPRE (a kind gift from M. Tanenbaum's group, Hubrecht Institute, Utrecht, The Netherlands) (Boersma et al., 2019), cut with EcoRI/XbaI and inserted into pcDNA3. The anti-HA_scFv frankenbodies have been described before (Zhao et al., 2019). For the anti-ALFA_Nb (Götzke et al., 2019), either sfGFP-GB1 or mEGFP were PCR amplified and inserted at the BamHI/NotI site of pNT-NAM01 pCMV-NbALFA-MCS (kindly provided by S. Frey, NanoTag Biotechnologies, Göttingen, Germany).

For *Drosophila* expression, pUASTLOTattB_anti-HA_fb_GFP was generated by cutting the frankenbody anti-HA-scFvX15F11_mEGFP (Zhao et al., 2019) with XhoI/XbaI and inserting the frankenbody into pUASTLOTattB (Kanca et al., 2014). For pUASTLOTattB_deGradHA, vhhGFP4 of pUAST_NSlmb-vhhGFP4 (Addgene #35575; Caussinus et al., 2011) was cut out and replaced with anti-HA_fb amplified by PCR. The resulting plasmid was cut with EcoRI/XbaI to insert deGradHA into pUASTLOTattB. For RIVwhite_TkvHAeGFP, the last two exons of Tkv and the intervening intron were cloned into the RIVwhite vector (Baena-Lopez et al., 2013), including sequences coding for one copy of the HA tag and eGFP prior to the stop codon.

All constructs were verified by sequencing. A schematic representation of the fusion constructs is provided in Fig. 1 and the amino acid sequences of the small tag binders used in this study are listed in Table S1.

### Cell cultures, transfections and imaging

HeLa S3α cells, kindly provided by D. Buser (Biozentrum, University of Basel, Switzerland), were maintained in Dulbecco's modified Eagle's medium supplemented with 10% fetal calf serum, 100 IU penicillin and 100 µg streptomycin per ml and routinely tested for mycoplasm contamination. One day before transfection, cells were seeded on glass cover slips placed into a 24-well plate at a density of 50,000-100,000 cells/well.

Transfections were carried out with 1 µg of total DNA (500 ng for each construct or with empty expression plasmid) and 3 µl of FuGENE HD Transfection Reagent (Promega), according to the manufacturer's instructions. 24 h post-transfection, cells were fixed in 4% paraformaldehyde, stained with Hoechst 33342 (Invitrogen) and mounted on standard microscope slides with VECTASHIELD.

Confocal images were acquired with a Leica point scanning confocal SP5-II-MATRIX microscope (Imaging Core Facility, Biozentrum, University of Basel) with a 63× HCX PLAN APO lambda blue objective and 1-2× zoom.

### *Drosophila* lines

Transgenic *Drosophila* lines carrying a UASTLOT_anti-HA_fb_GFP or UASTLOT_deGradHA insertion in chromosomal position Chr3L, 68A4 (attP2) were generated by standard procedures using PhiC31/attB-mediated integration. Flies carrying genome-engineered Tkv containing one copy of the HA tag followed by eGFP at the C-terminus were generated using previously described methods (Baena-Lopez et al., 2013). We used previously described *tkv*[ko,attP] containing flies, in which the last two exons of the gene were replaced by an attP-containing cassette (Norman et al., 2016). The missing exons were reconstituted by standard PhiC31/attB transgenesis using RIVwhite_TkvHAeGFP.

Flies containing UAS_NSlmb-vhhGFP4 (deGradFP), *tkv*[ko,attP] (*tkv*[def]) or Tkv3×HA have been previously described (Caussinus et al., 2011; Norman et al., 2016; Tracy Cai et al., 2019). UASpH2Av::Flag-HA (H2Av-Flag-HA) flies were a kind gift from N. Iovino's group (Max Planck Institute for Immunobiology and Epigenetics, Freiburg, Germany). UASHistone4-3×HA (H4-3×HA) flies were created by the FlyORF Zurich ORFeome Project (Bischof et al., 2013) (Fly Line ID F000777). Brk-GAL4 (53707), GMR-GAL4 (1104), UASYkiS168A-HA-eGFP (28836; described by Oh and Irvine, 2008) and UASGFP (4776) flies were provided by the Bloomington *Drosophila* Stock Center. Ap-GAL4 flies were originally obtained from W. Gehring (Biozentrum, University of Basel, Switzerland).

### Immunohistochemistry and imaging of *Drosophila* samples

Salivary glands and wing imaginal discs from 3rd instar *Drosophila* larvae were dissected, fixed and stained using standard procedures. The following antibodies were used: rabbit anti-GFP (1:500, Abcam), rat anti-HA (1:200, Roche), mouse anti-Flag (1:500, Sigma), rabbit anti-Sal (1:500, R. Barrio), rabbit anti-pSmad3 (1:500, Abcam), Alexa fluorophore-conjugated secondary antibodies (1:500; A11031, A11034, A11036, A11077) and Hoechst 33342 (1:5000; Invitrogen). Images were acquired using a Zeiss LSM880 laser scanning confocal microscope (Life Imaging Center, Center for Biological Systems Analysis, University of Freiburg). For quantification, identical sized and positioned boxes parallel to the dorsoventral compartment boundary were generated in the ventral (control) and dorsal (experimental) compartments (see Fig. 8B) and average pixel intensity over length was measured using the plot profile function in ImageJ. Plot values were transferred to Excel (Microsoft) and averaged over 20 consecutive values for the generation of the intensity profile plots.

## Supplementary Material

Supplementary information

Reviewer comments
