# Supplementary material for: Protein manipulation using single copies of short peptide tags in cultured cells and in Drosophila melanogaster
Source: Development. 2021 Mar 16;148(6):dev191700. doi: 10.1242/dev.191700 (PMC7990863; doi:10.1242/dev.191700)
Supplement: Supplementary information [file develop-148-191700-s1.pdf]

Figure S1A

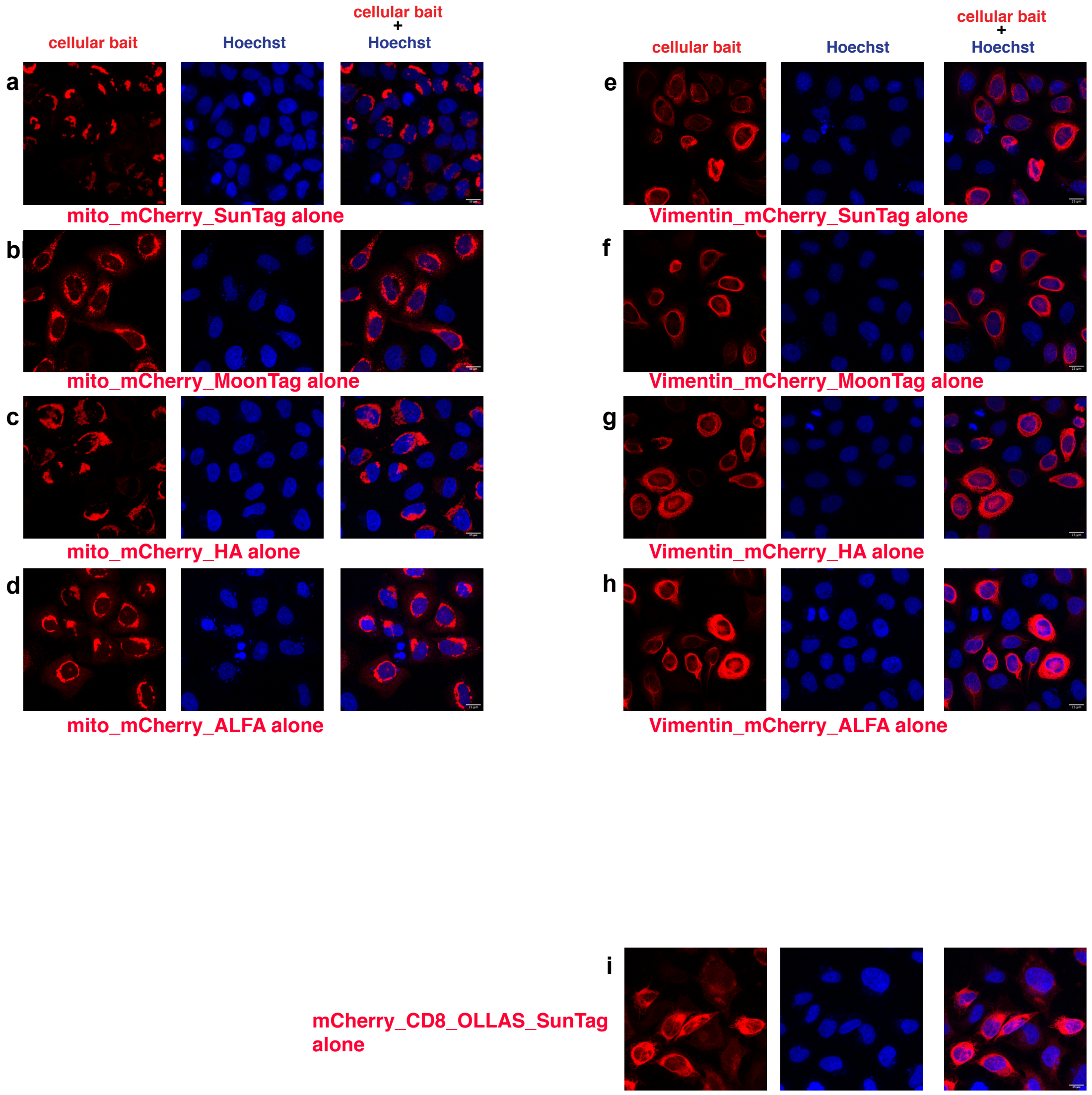

Figure S1A. Intracellular expression of mitochondrial, filament and membrane baits
Confocal images of HeLa cells transiently transfected with **(a)** mito_mCherry_SunTag, **(b)** mito_mCherry_MoonTag, **(c)** mito_mCherry_HA, **(d)** mito_mCherry_ALFA, **(e)** Vimentin_mCherry_SunTag, (**f**) Vimentin_mCherry_MoonTag, (**g**) Vimentin_mCherry_HA, (**h**) Vimentin_mCherry_ALFA, (**i**) mCherry_CD8_OLLAS_SunTag. The first column of each row indicated by the letter represents the mCherry channel (red), the second column is the nuclear Hoechst staining (blue) and the third column is the overlay of the two channels channels (with the scale bar in white (15 μm)), showing the localization of the mitochondrial (**a-d**), filaments (**e-h**) and membrane (**i**) baits. Images were taken 24 hours post transfection. Transfected constructs are indicated at bottom of each row and the single and merge channels are indicated at the top of the respective columns. The figures are from a representative experiment, performed at least three times.

Development • Supplementary information

Figure S1B

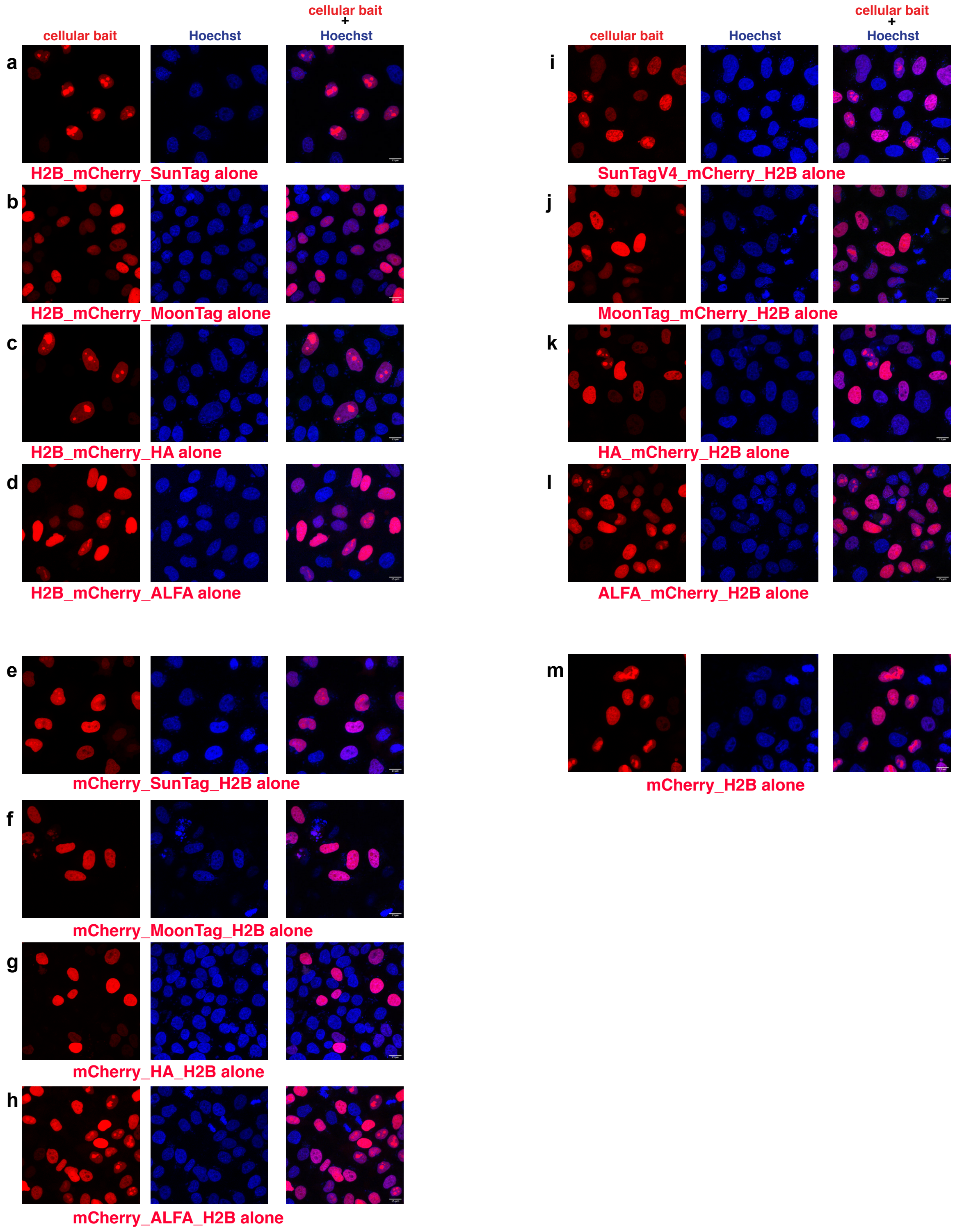

Development • Supplementary information

Figure S1B. Intracellular expression of the nuclear baits

Confocal images of HeLa cells transiently transfected with **(a)** H2B_mCherry_SunTag, **(b)** H2B_mCherry_MoonTag, **(c)** H2B_mCherry_HA, **(d)** H2B_mCherry_ALFA, **(e)** mCherry_SunTag_H2B, (**f**) mCherry_MoonTag_H2B, (**g**) mCherry_HA_H2B, (**h**) mCherry_ALFA_H2B, (**i**) SunTag_mCherry_H2B, (**j**) MoonTag_mCherry_H2B, (**k**) HA_mCherry_H2B, (**l**) ALFA_mCherry_H2B, (**m**) mCherry_H2B. The first column of each row indicated by the letter represents the mCherry channel (red), the second column is the nuclear Hoechst staining (blue) and the third column is the overlay of the two channels channels (with the scale bar in white (15 μm)), showing the localization of the nuclear baits. Images were taken 24 hours post transfection. Transfected constructs are indicated at bottom of each row and the single and merge channels are indicated at the top of the respective columns. The figures are from a representative experiment, performed at least three times.

Figure S2

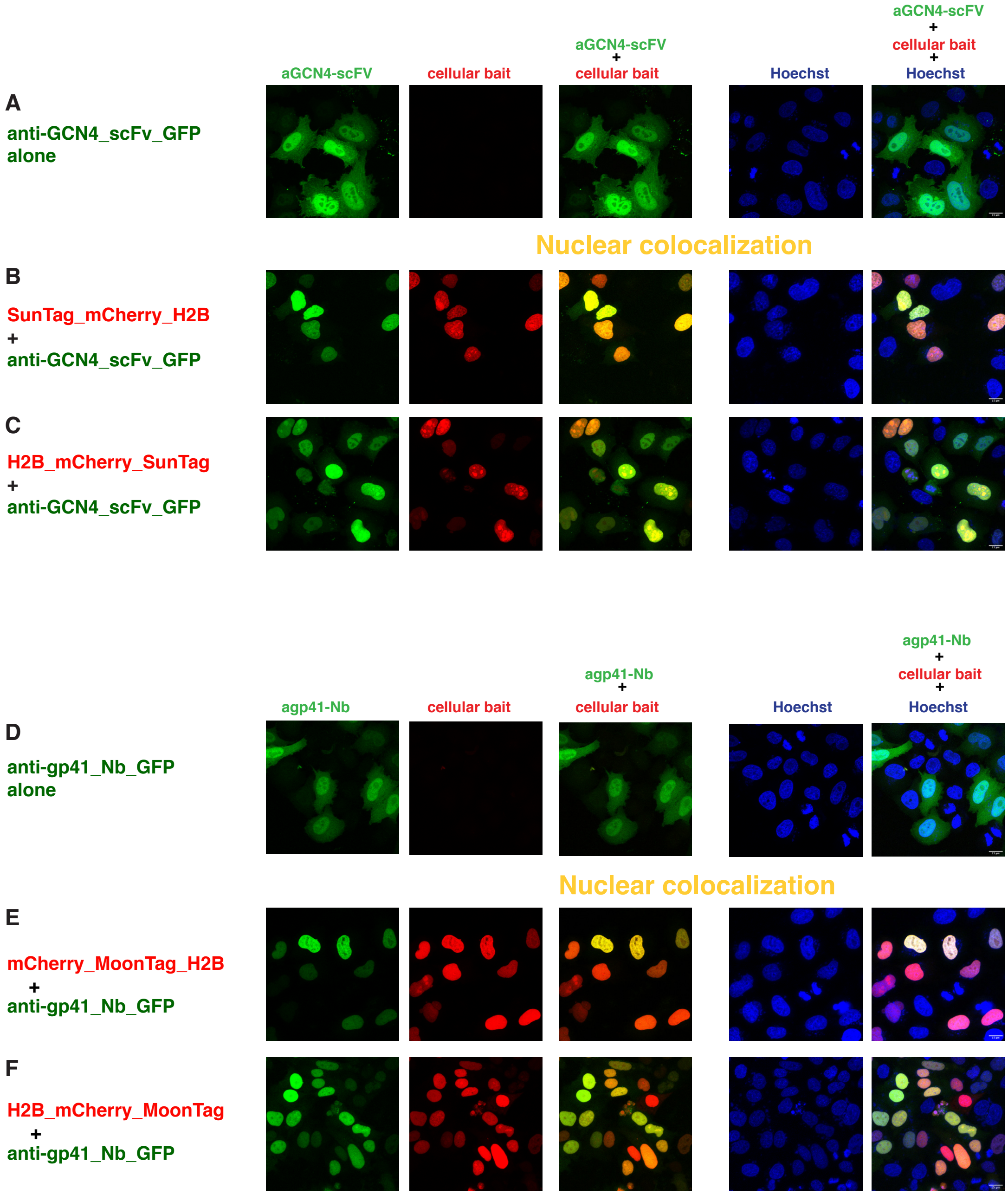

Development • Supplementary information

Figure S2. Intracellular binding of anti-GCN4_scFv_GFP and anti-gp41_Nb_GFP to nuclear baits Confocal images of HeLa cells transiently transfected with **(A)** anti-GCN4_scFv_GFP alone; the combination of anti-GCN4_scFv_GFP and **(B)** SunTag_mCherry_H2B; **(C)** H2B_mCherry_SunTag; **(D)** anti-gp41_Nb_GFP alone; the combination of anti-gp41_Nb_GFP and **(E)** mCherry_MoonTag_H2B; **(F)** H2B_mCherry_MoonTag. The first column represents the GFP channel (green), the second column is the mCherry channel (red), the third column is the overlay of the two channels, showing the colocalization (indicated in yellow) of the antiGCN4_scFv (**A-C**) or the anti-gp41_Nb (**D-F**) with the respectively tagged nuclear baits; the fourth column represents the nuclear Hoechst staining (blue) and the fifth column is the merge of all three channels (with the scale bar in white (15 μm) on the bottom right corner). Images were taken 24 hours post transfection. Transfected constructs are indicated at the left of each row and the single and merge channels are indicated at the top of the respective columns. The figures are from a representative experiment, performed at least three times.

Figure S3

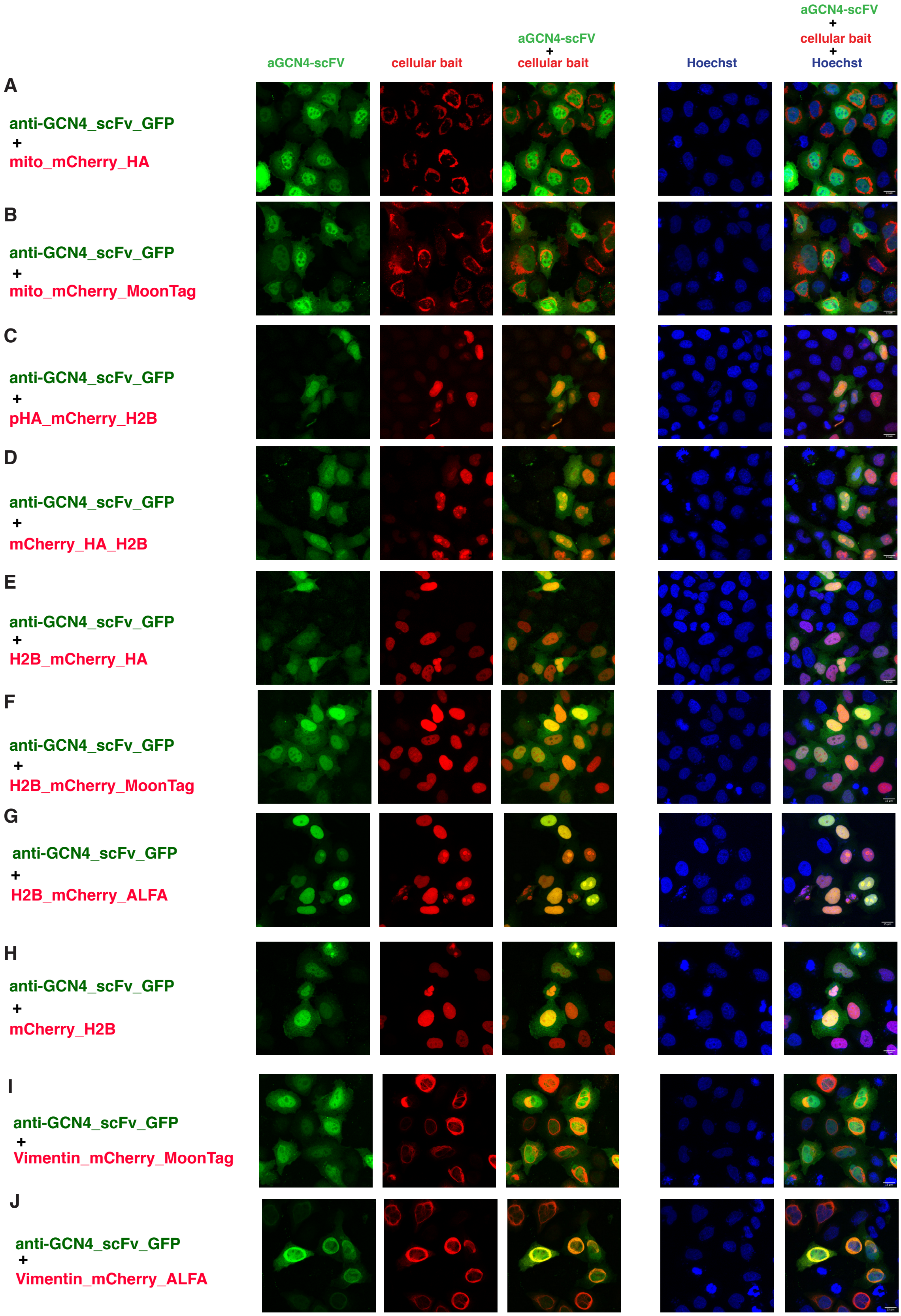

Development • Supplementary information

Figure S3. Negative controls of anti-GCN4_scFv_GFP

Confocal images of HeLa cells transiently transfected with the combination of anti-GCN4_scFv_GFP and **(A)** mito_mCherry_HA, (**B**) mito_mCherry_MoonTag, **(C)** HA_mCherry_H2B, **(D)** mCherry_HA_H2B, **(E)** H2B_mCherry_HA, (**F**) H2B_mCherry_MoonTag, (**G**) H2B_mCherry_ALFA, (**H**) mCherry_H2B, (**I**) Vimentin_mCherry-MoonTag, (**J**) Vimentin_mCherry_ALFA. The first column represents the GFP channel (green), the second column is the mCherry channel (red), the third column is the overlay of the two channels, showing the colocalization (indicated in yellow) of the anti-GCN4_scFv with mitochondrial (**A-B**), nuclear (**C-H**),) and filaments (**I-J**) baits carrying different tags; the fourth column represents the nuclear Hoechst staining (blue) and the fifth column is the merge of all three channels (with the scale bar in white (15 μm) on the bottom right corner). Images were taken 24 hours post transfection. Transfected constructs are indicated at the left of each row and the single and merge channels are indicated at the top of the respective columns. The figures are from a representative experiment.

Development • Supplementary information

Figure S4

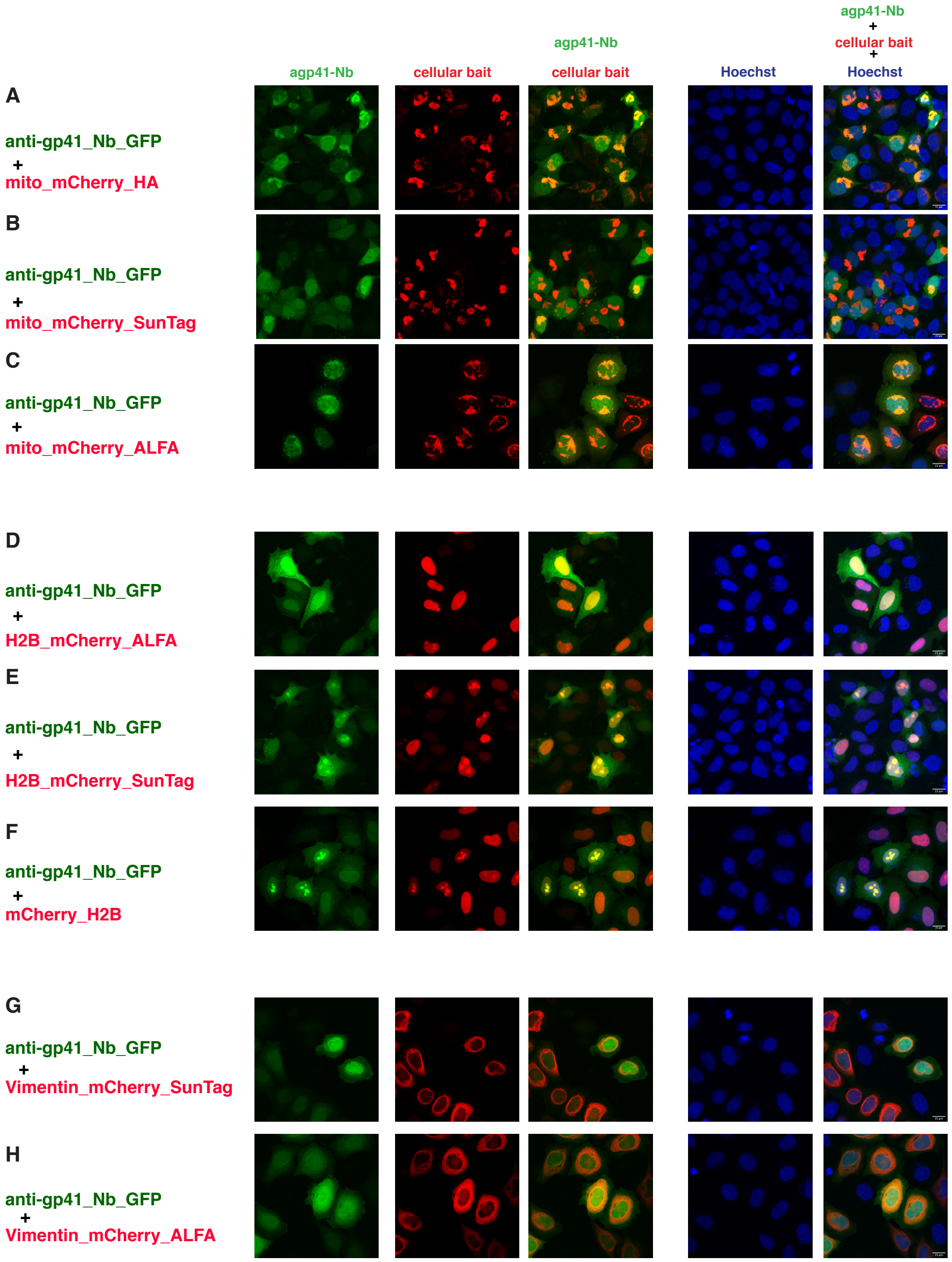

Development • Supplementary information

Figure S4. Negative controls of anti-gp41_Nb_GFP

Confocal images of HeLa cells transiently transfected with the combination of anti-gp41_Nb_GFP and **(A)** mito_mCherry_HA, (**B**) mito_mCherry_SunTag, **(C)** mito_mCherry_ALFA **(D)** H2B_mCherry_ALFA, **(E)** H2B_mCherry_SunTag, (**F)** mCherry_H2B, (**G**) Vimentin_mCherry-SunTag, (**H**) Vimentin_mCherry_ALFA. The first column represents the GFP channel (green), the second column is the mCherry channel (red), the third column is the overlay of the two channels, showing the colocalization (indicated in yellow) of the anti-gp41_Nb with mitochondrial (**A-C**), nuclear (**D-F**), and filaments (**G-H**) baits carrying different tags; the fourth column represents the nuclear Hoechst staining (blue) and the fifth column is the merge of all three channels (with the scale bar in white (15 µm) on the bottom right corner). Images were taken 24 hours post transfection. Transfected constructs are indicated at the left of each row and the single and merge channels are indicated at the top of the respective columns. The figures are from a representative experiment.

Development • Supplementary information

Figure S5

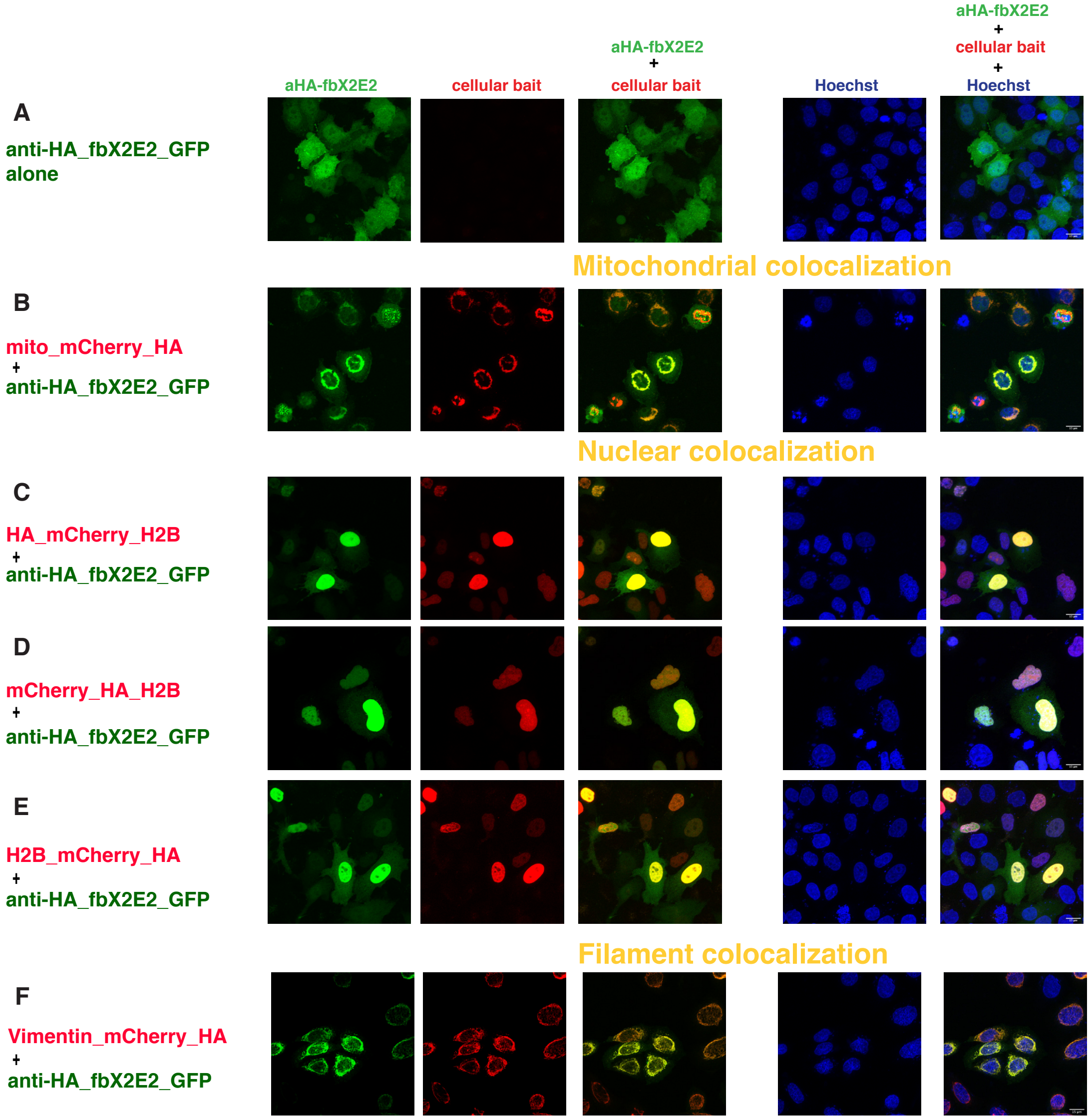

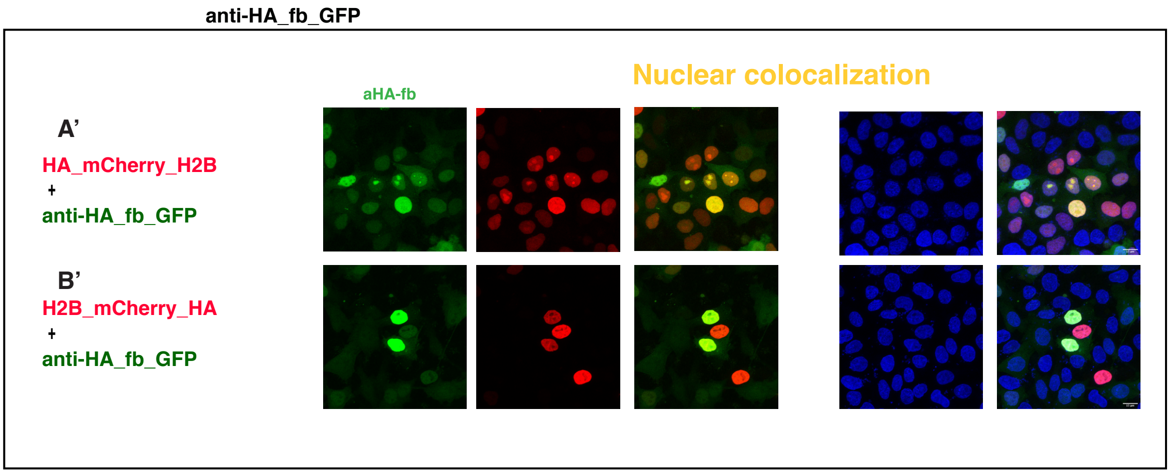

Development • Supplementary information

Figure S5. Intracellular binding of anti-HA_fbX2E2_GFP and extra nuclear colocalization of anti-HA_fb_GFP (HA system)

Confocal images of HeLa cells transiently transfected with **(A)** anti-HA_fbX2E2_GFP alone; the combination of anti-HA_fbX2E2_GFP and **(B)** mito_mCherry_HA; **(C)** HA_mCherry_H2B; **(D)** mCherry_HA_H2B; (**E**) H2B_mCherry_H2B; (**F**) Vimentin_mCherry_HA. The confocal images in lower black frame represent the cotransfection of anti-HA_fb_GFP with (**A'**) HA_mCherry_H2B or (**B'**) H2B_mCherry_HA.The first column represents the GFP channel (green), the second column is the mCherry channel (red), the third column is the overlay of the two channels, showing the colocalization (indicated in yellow) of the anti-HA_scFvs with the respective mitochondrial (**B**), nuclear (**C-E**, **A'-B'**) and filaments (**F**) baits; the fourth column represents the nuclear Hoechst staining (blue) and the fifth column is the merge of all three channels (with the scale bar in white (15 μm) on the bottom right corner). Images were taken 24 hours post transfection. Transfected constructs are indicated at the left of each row and the single and merge channels are indicated at the top of the respective columns. The figures are from a representative experiment, performed at least three times.

Development • Supplementary information

Figure S6

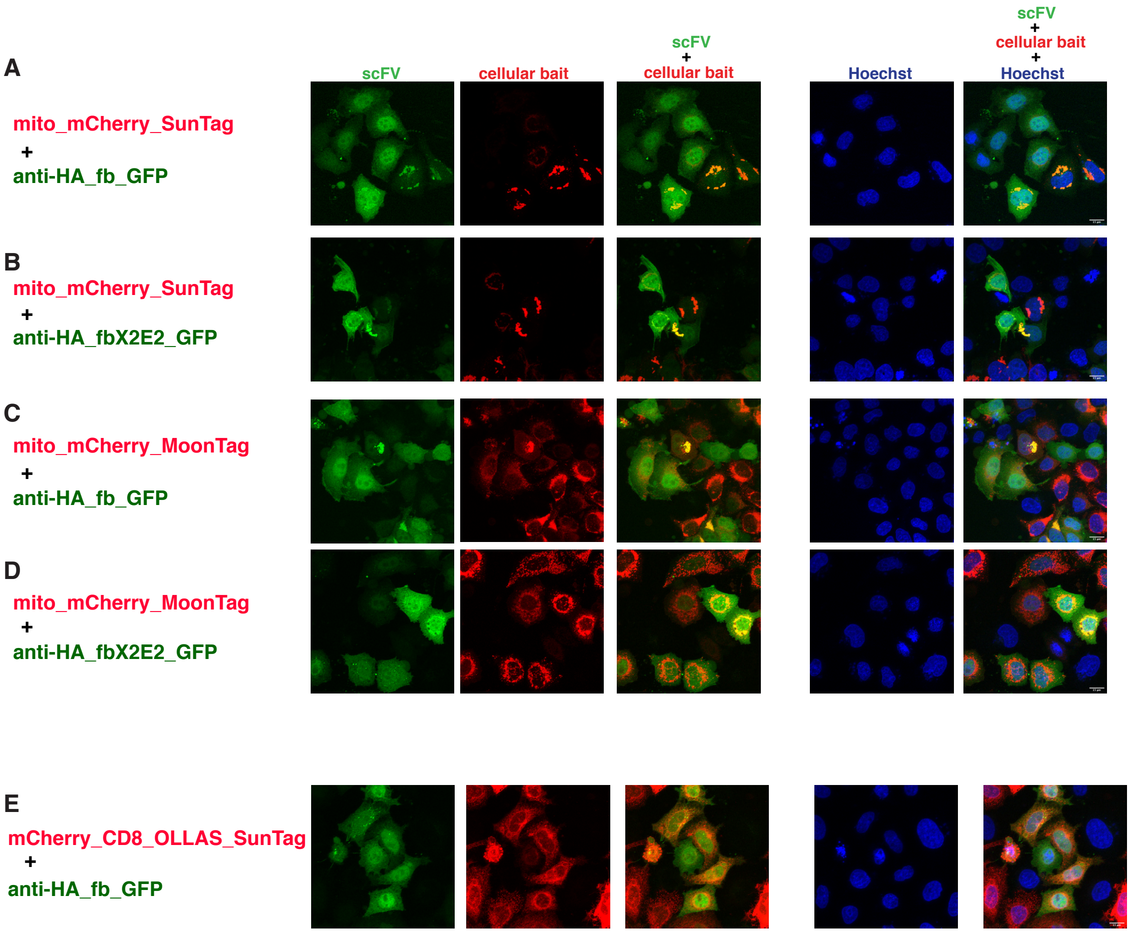

Figure S6. Negative mitochondrial and membrane controls of anti-HA_scFvs

Confocal images of HeLa cells transiently transfected with the combination of anti-HA_fb_GFP (**A**, **C** ,**E**) or anti-HA_fbX2E2_GFP (**B**, **D**) and **(A-B)** mito_mCherry_SunTag, **(C-D)** mito_mCherry_MoonTag, (**E**) mCherry_CD8_OLLAS_SunTag. The first column represents the GFP channel (green), the second column is the mCherry channel (red), the third column is the overlay of the two channels, showing the colocalization (indicated in yellow) of the anti-HA_scFvs with mitochondrial (**A-D**), and membrane (**E**) baits carrying different tags; the fourth column represents the nuclear Hoechst staining (blue) and the fifth column is the merge of all three channels (with the scale bar in white (15 μm) on the bottom right corner). Images were taken 24 hours post transfection. Transfected constructs are indicated at the left of each row and the single and merge channels are indicated at the top of the respective columns. The figures are from a representative experiment.

Development • Supplementary information

Figure S7

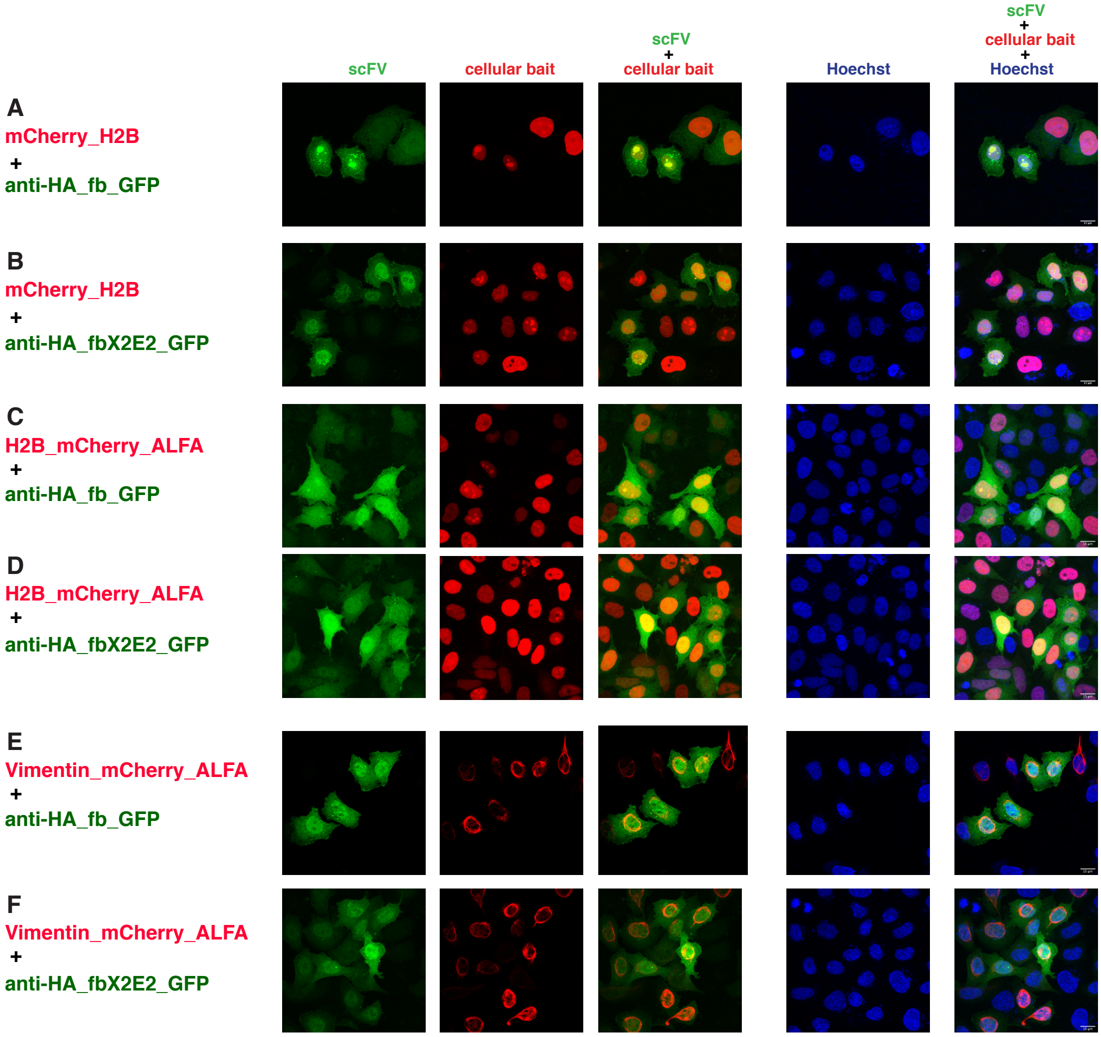

Figure S7. Negative nuclear and filaments controls of anti-HA_scFvs

Confocal images of HeLa cells transiently transfected with the combination of anti-HA_fb_GFP (**A, C, E**) or anti-HA_fbX2E2_GFP (**B, D, F**) and **(A-B)** mCherry_H2B, (**C-D**) H2B_mCherry_ALFA, **(E-F)** Vimentin_mCherry_ALFA. The first column represents the GFP channel (green), the second column is the mCherry channel (red), the third column is the overlay of the two channels, showing the colocalization (indicated in yellow) of the anti-HA_scFvs with nuclear (**A-D**), and filaments (**E-F**) baits carrying different tags; the fourth column represents the nuclear Hoechst staining (blue) and the fifth column is the merge of all three channels (with the scale bar in white (15 µm) on the bottom right corner). Images were taken 24 hours post transfection. Transfected constructs are indicated at the left of each row and the single and merge channels are indicated at the top of the respective columns. The figures are from a representative experiment.

Development • Supplementary information

Figure S8

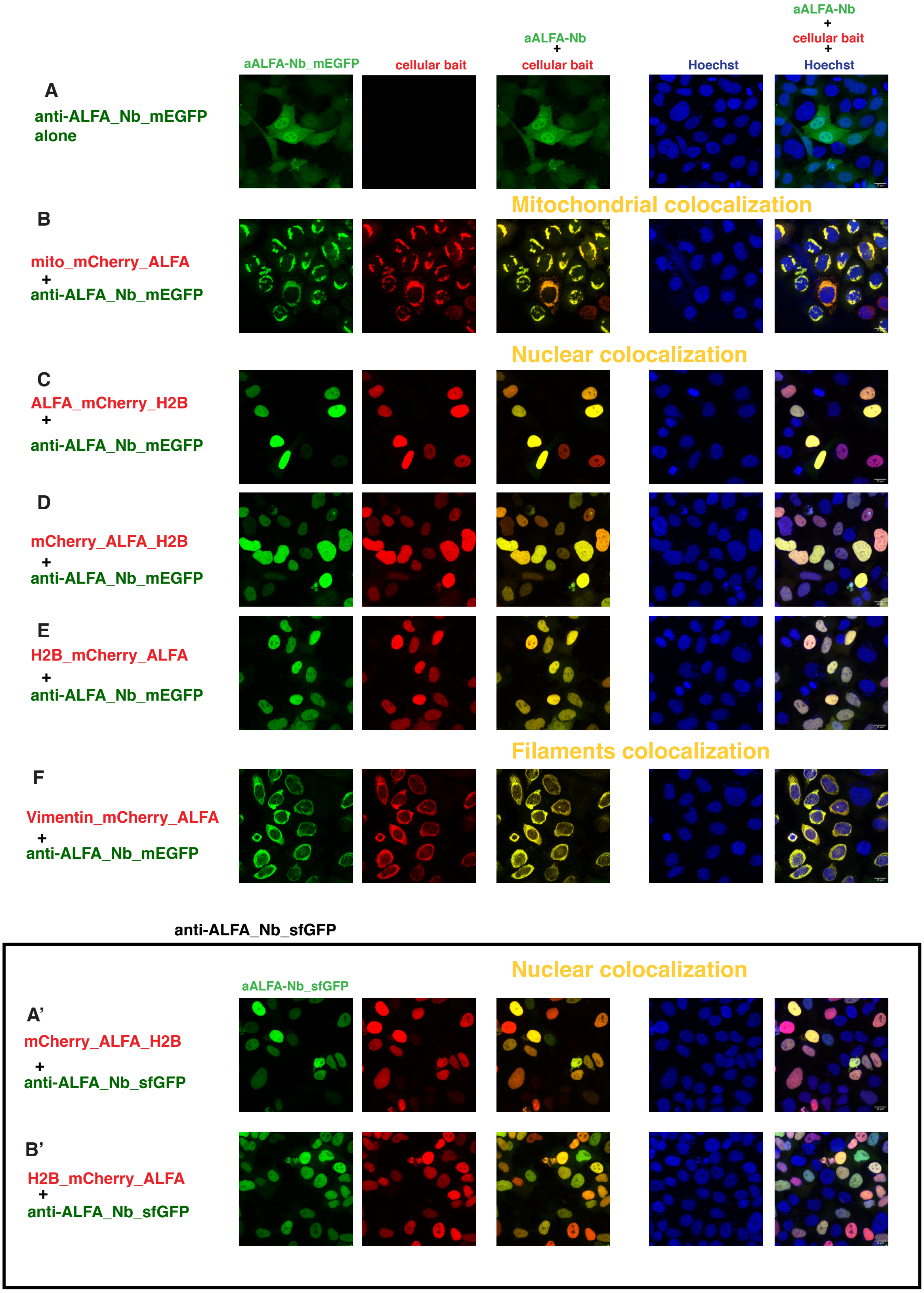

Development • Supplementary information

Figure S8. Intracellular binding of anti-ALFA_Nb_mEGFP and extra nuclear colocalization of anti-ALFA_Nb_sfGFP

Confocal images of HeLa cells transiently transfected with **(A)** anti-ALFA_Nb_mEGFP alone; the combination of anti-ALFA_Nb_mEGFP and **(B)** mito_mCherry_ALFA; **(C)** ALFA_mCherry_H2B; **(D)** mCherry_ALFA_H2B; (**E**) H2B_mCherry_ALFA; (**F**) Vimentin_mCherry_ALFA. The confocal images in lower black frame represent the cotransfection of anti-ALFA_Nb_sfGFP with (**A'**) mCherry_ALFA_H2B or (**B'**) H2B_mCherry_ALFA.The first column represents the GFP channel (green), the second column is the mCherry channel (red), the third column is the overlay of the two channels, showing the colocalization (indicated in yellow) of the anti-ALFA Nanobodies with the respective mitochondrial (**B**), nuclear (**C-E**, **A'-B'**) and filaments (**F**) baits; the fourth column represents the nuclear Hoechst staining (blue) and the fifth column is the merge of all three channels (with the scale bar in white (15 μm) on the bottom right corner). Images were taken 24 hours post transfection. Transfected constructs are indicated at the left of each row and the single and merge channels are indicated at the top of the respective columns. The figures are from a representative experiment, performed at least three times.

Development • Supplementary information

Figure S9A

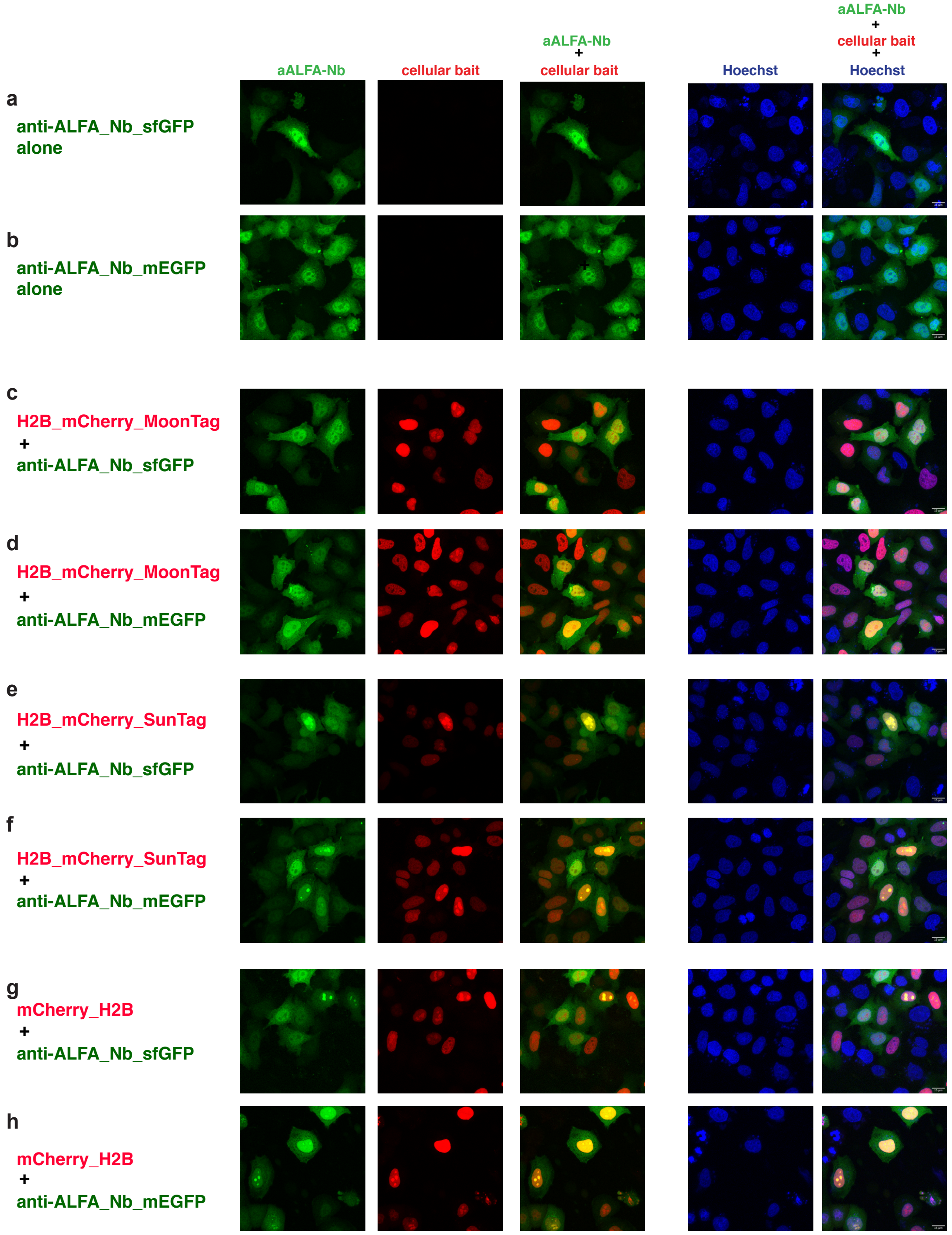

Development • Supplementary information

Figure S9A. Negative nuclear controls of anti-ALFA nanobodies

Confocal images of HeLa cells transiently transfected with anti-ALFA_Nb_sfGFP (**a, c, e** and **g**) or anti-ALFA_Nb_mEGFP (**b, d, f** and **h**)_alone **(a-b)** or in combination with (**c-d**) H2B_mCherry_MoonTag, **(e-f)** H2B_mCherry_SunTag, (**G-H**) mCherry_H2B. The first column represents the GFP channel (green), the second column is the mCherry channel (red), the third column is the overlay of the two channels, showing the colocalization (indicated in yellow) of the anti-ALFA nanobody with nuclear (**c-h**) baits carrying different tags; the fourth column represents the nuclear Hoechst staining (blue) and the fifth column is the merge of all three channels (with the scale bar in white (15 μm) on the bottom right corner). Images were taken 24 hours post transfection. Transfected constructs are indicated at the left of each row and the single and merge channels are indicated at the top of the respective columns. The figures are from a representative experiment.

Development • Supplementary information

Figure S9B

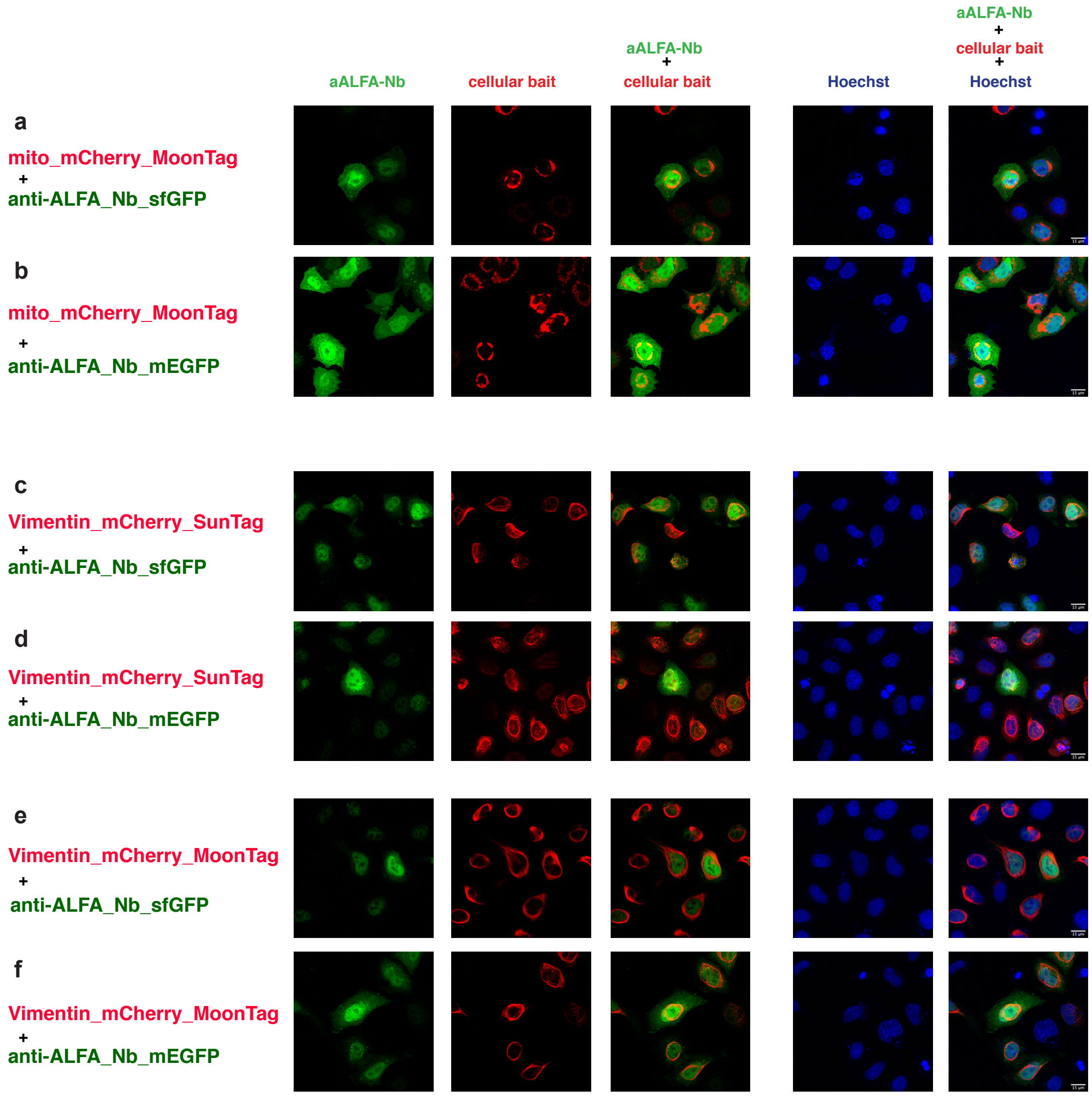

Figure S9B. Negative mitochondrial and filaments controls of anti-ALFA nanobodies

Confocal images of HeLa cells transiently transfected with the combination of anti-ALFA_Nb_sfGFP (**a, c, e**) or anti-ALFA_Nb_mEGFP (**b, d, f**) and **(a-b)** mito_mCherry_MoonTag, (**c-d**) Vimentin_mCherry_SunTag, **(e-f)** Vimentin_mCherry_MoonTag1. The first column represents the GFP channel (green), the second column is the mCherry channel (red), the third column is the overlay of the two channels, showing the colocalization (indicated in yellow) of the anti-ALFA nanobodies with mitochondrial (**a-b**), and filaments (**c-f**) baits carrying different tags; the fourth column represents the nuclear Hoechst staining (blue) and the fifth column is the merge of all three channels (with the scale bar in white (15 μm) on the bottom right corner). Images were taken 24 hours post transfection. Transfected constructs are indicated at the left of each row and the single and merge channels are indicated at the top of the respective columns. The figures are from a representative experiment.

Development • Supplementary information

Figure S10

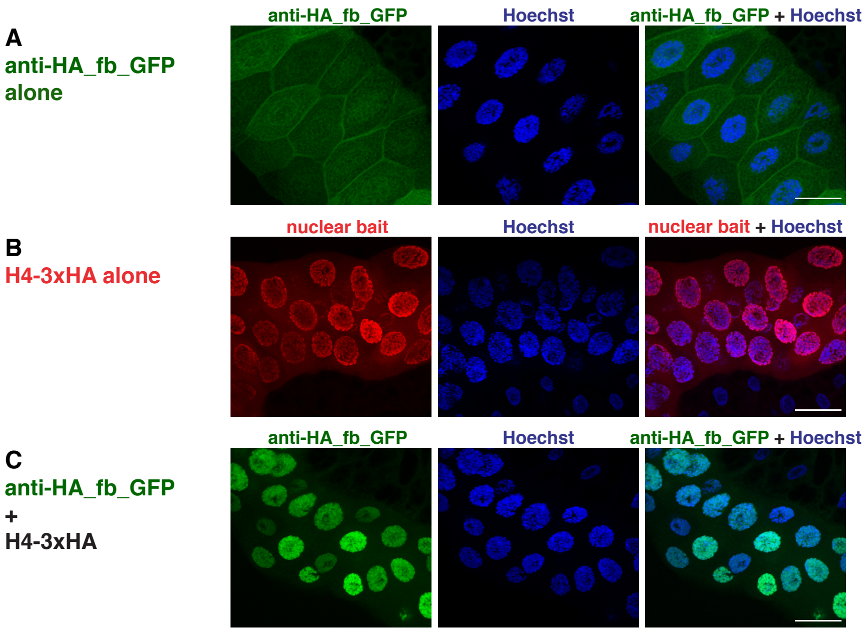

Figure S10. Intracellular binding of anti-HA_fb_GFP (HA system) *in vivo*

Confocal images of *Drosophila* larval salivary glands expressing anti-HA_fb_GFP alone (**A**), the nuclear bait H4-3xHA alone (**B**), or a combination of anti-HA_fb_GFP and H4-3xHA (**C**). The first column represents the GFP channel (green, **A** and **C**) or the anti-HA staining channel (red, **B**). The second column represents the nuclear Hoechst staining (blue) and the third column is the merge of the two respective channels. Scale bars are 50 µm. Salivary glands were obtained from third instar *Drosophila* larvae expressing the UAS constructs indicated at the left of each row using brk-GAL4 as a driver. Single and merged channels are indicated at the top of the respective channel.

Development • Supplementary information

Figure S11

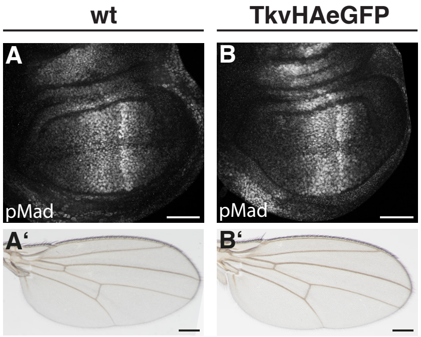

Figure S11. Validation of TkvHAeGFP activity

Confocal images of pMad distribution in 3rd instar larval wing imaginal discs and adult wings of wild-type flies (**A and A'**) or flies homozygous for the generated *tkvHAeGFP* allele (**B and B'**). Scale bars are 50 µm (larval discs) and 200 µm (adult wings).

Development • Supplementary information

Figure S12

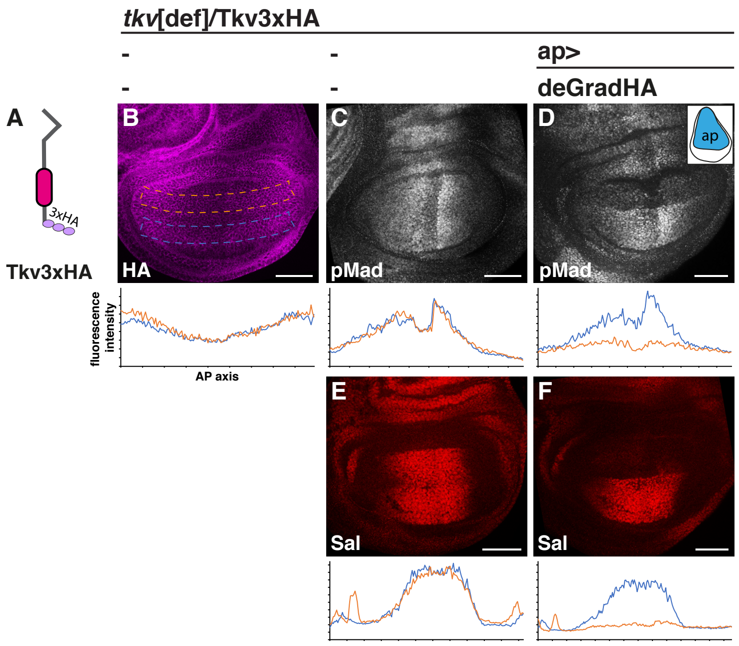

Figure S12. Manipulation of endogenously 3xHA-tagged proteins by deGradHA

Schematic representation (**A**) and wing disc expression (**B**) of an endogenously tagged Tkv version with three copies of HA. Confocal images of pMad and Sal distribution in 3rd instar wing imaginal discs of larvae carrying the *tkv3xHA* allele over a *tkv* chromosomal deficiency (**C, E**) or larvae which additionally express deGradHA under the control of ap-GAL4 in the dorsal compartment (**D, F**). Plots below each panel depict relative fluorescent intensity of ventral (control, blue) and dorsal (experimental, orange) cells along the AP axis of the wing pouch (coloured boxes in panel **B** indicate areas used for quantification). Note the strong reduction of pMad and the complete absence of Sal in dorsal cells in panels **D** and **F**, respectively. Scale bars are 50 µm.

Table S1

**peptide binders construct**	**aa sequence** (color scheme as in Fig.1)
anti-GCN4_scFv_GFP	MGPDIVMTQSPSSLSASVGDRVTITCRSSTGAVTTSNYASWVQEKPGKLFKGLIGGTNNRAPGVPSRFSGSLIGDKATLTISSLQPEDFATYFCALWYSNHW<br>VFGQGTKVELKRGGGGSGGGGSGGGGSSGGGSEVKLLESGGGLVQPGGSLKLSCAVSGFSLTDYGVNWVRQAPGRGLEWIGVIWGDGITDYNSALKDR<br>FIISKDNGKNTVYLQMSKVRSDDTALYYCVTGLFDYWGQGTLVTVSSYPYDVPDYAGGGGGSGGGGSGGGGSGGGGSLDPGGGGSGSKGEELFTGVVPI<br>LVELDGDVNGHKFSVRGEGEGDATNGKLTLKFICTTGKLPVPWPTLVTTLTYGVQCFSRYPDHMKRHDFFKSAMPEGYVQERTISFKDDGTYKTRAEVKFEG<br>DTLVNRIELKGIDFKEDGNILGHKLEYNFNSHNVYITADKQKNGIKANFKIRHNVEDGSVQLADHYQQNTPIGDGPVLLPDNHYLSTQSVLSKDPNEKRDHMVL<br>LEFVTAAGITHGMDELYKGGGRTEEYKLILNGKTLKGETTTEAVDAATAEKVFKQYANDNGVDGEWTYDDATKTFTVTE*
anti-gp41_Nb_GFP	MEVQLVESGGGLVQPGGSLRLSCAASGSISSVDVMSWYRQAPGKQRELVAFITDRGRTNYKVSVKGRFTISRDNSKNMVYLQMNSLKPEDTADYLCRAES<br>RTSWSSPSPLDVWGRGTQVTVSSLDPGGGGSGSKGEELFTGVVPILVELDGDVNGHKFSVRGEGEGDATNGKLTLKFICTTGKLPVPWPTLVTTLTYGVQCF<br>SRYPDHMKRHDFFKSAMPEGYVQERTISFKDDGTYKTRAEVKFEGDTLVNRIELKGIDFKEDGNILGHKLEYNFNSHNVYITADKQKNGIKANFKIRHNVEDGS<br>VQLADHYQQNTPIGDGPVLLPDNHYLSTQSVLSKDPNEKRDHMVLLEFVTAAGITHGMDELYKGGGRTEEYKLILNGKTLKGETTTEAVDAATAEKVFKQYAN<br>DNGVDGEWTYDDATKTFTVTE*
anti-HA_fb_GFP	MAEVKLVESGGGLVKPGGSLKLSCAASGFTFSSYGMSWVRQTPEKRLEWVATISRGGSYTYYPDSVKGRFTISRDNAKNTLYLQMSSLRSEDTAIYYCARRE<br>TYDEKGFAYWGQGTTLTVSSGGGGSGGGGSGGGGSDIVLTQSPASLTVSLGQRATISCKSSQSLLNSGNQKNYLTWYQQKPGQPPKLLIYWASTRESGIPA<br>RFSGSGSGTDFTLNIHPVEEEDAATYYCQNDNSHPLTFGAGTKLEIKRAAAKGEFGGGGSGGGGSGGGGSGGGGSGGGGSGSKGEELFTGVVPILVELDG<br>DVNGHKFSVSGEGEGDATYGKLTLKFICTTGKLPVPWPTLVTTLTYGVQCFSRYPDHMKQHDFFKSAMPEGYVQERTIFFKDDGNYKTRAEVKFEGDTLVNRI<br>ELKGIDFKEDGNILGHKLEYNYNSHNVYIMADKQKNGIKVNFKIRHNIEDGSVQLADHYQQNTPIGDGPVLLPDNHYLSTQSKLSKDPNEKRDHMVLLEFVTAA<br>GITLGMDELYK*
anti-HA_fbX2E2_GFP	MAEVQLVESGGDLVKPGGSLKLSCAASGFTFSSYGMSWVRQTPDKRLEWVATISRGGSYTYYPDSVKGRFTISRDNAKNTLYLQMSSLKSEDTAMYYCARR<br>ETYDEKGFAYWGQGTSVTVSSGGGGSGGGGSGGGGSDIVLTQSPASLAVSLGQRATISCKSSQSLLNSGNQKNYLTWYQQKPGQPPKLLIYWASTRESGIP<br>ARFSGSGSGTDFTLNIHPVEEEDAATYYCQNDNSHPLTFGGGTKLEIKRAAAKGEFGGGGSGGGGSGGGGSGGGGSGGGGSGSKGEELFTGVVPILVELD<br>GDVNGHKFSVSGEGEGDATYGKLTLKFICTTGKLPVPWPTLVTTLTYGVQCFSRYPDHMKQHDFFKSAMPEGYVQERTIFFKDDGNYKTRAEVKFEGDTLVN<br>RIELKGIDFKEDGNILGHKLEYNYNSHNVYIMADKQKNGIKVNFKIRHNIEDGSVQLADHYQQNTPIGDGPVLLPDNHYLSTQSKLSKDPNEKRDHMVLLEFVT<br>AAGITLGMDELYK*
anti-ALFA_Nb_sfGFP	MGSGDASDSEVQLQESGGGLVQPGGSLRLSCTASGVTISALNAMAMGWYRQAPGERRVMVAAVSERGNAMYRESVQGRFTVTRDFTNKMVSLQMDN<br>LKPEDTAVYYCHVLEDRVDSFHDYWGQGTQVTVSSEPKTPKPQTSGSSKGEELFTGVVPILVELDGDVNGHKFSVRGEGEGDATNGKLTLKFICTTGKLPVPW<br>PTLVTTLTYGVQCFSRYPDHMKRHDFFKSAMPEGYVQERTISFKDDGTYKTRAEVKFEGDTLVNRIELKGIDFKEDGNILGHKLEYNFNSHNVYITADKQKNGIK<br>ANFKIRHNVEDGSVQLADHYQQNTPIGDGPVLLPDNHYLSTQSVLSKDPNEKRDHMVLLEFVTAAGITHGMDELYKGGGRTEEYKLILNGKTLKGETTTEAVD<br>AATAEKVFKQYANDNGVDGEWTYDDATKTFTVTE*
anti-ALFA_Nb_mEGFP	MGSGDASDSEVQLQESGGGLVQPGGSLRLSCTASGVTISALNAMAMGWYRQAPGERRVMVAAVSERGNAMYRESVQGRFTVTRDFTNKMVSLQMDN<br>LKPEDTAVYYCHVLEDRVDSFHDYWGQGTQVTVSSEPKTPKPQTSGSSKGEELFTGVVPILVELDGDVNGHKFSVSGEGEGDATYGKLTLKFICTTGKLPVPW<br>PTLVTTLTYGVQCFSRYPDHMKQHDFFKSAMPEGYVQERTIFFKDDGNYKTRAEVKFEGDTLVNRIELKGIDFKEDGNILGHKLEYNYNSHNVYIMADKQKNG<br>IKVNFKIRHNIEDGSVQLADHYQQNTPIGDGPVLLPDNHYLSTQSKLSKDPNEKRDHMVLLEFVTAAGITLGMDELYK*
	Nslimb is shown in red
deGradHA	MMKMETDKIMDETNSNAQAFTTTMLYDPVRKKDSSPTYQTERELCFQYFTQWSESGQVDFVEHLLSRMCHYQHGQINAYLKPMLQRDFITLLPIKGLDHIAE<br>NILSYLDAESLKSSELVCKEWLRVISEGMLWKKLIERKVRTDSLWRGLAERRNWMQYLFKPRPGQTQRPHSFHRELFPKIMNDIDSIENNWRTGRHLERSTS<br>MAEVKLVESGGGLVKPGGSLKLSCAASGFTFSSYGMSWVRQTPEKRLEWVATISRGGSYTYYPDSVKGRFTISRDNAKNTLYLQMSSLRSEDTAIYYCARRE<br>TYDEKGFAYWGQGTTLTVSSGGGGSGGGGSGGGGSDIVLTQSPASLTVSLGQRATISCKSSQSLLNSGNQKNYLTWYQQKPGQPPKLLIYWASTRESGIPA<br>RFSGSGSGTDFTLNIHPVEEEDAATYYCQNDNSHPLTFGAGTKLEIKRATS*

Amino acid sequences of the fusion proteins containing the peptide binders of this study. Color codes are the same as in Figure 1. For deGradHA, the Nslimb part is shown in red.
