# Supplementary material for: Protein manipulation using single copies of short peptide tags in cultured cells and in Drosophila melanogaster
Source: Development. 2021 Mar 16;148(6):dev191700. doi: 10.1242/dev.191700 (PMC7990863; doi:10.1242/dev.191700)
Supplement: Reviewer comments [file develop-148-191700_review_history.pdf]

Protein manipulation using single copies of short peptide tags in cultured cells and in *Drosophila melanogaster*

M. Alessandra Vigano, Clara-Maria Ell, Manuela M. M. Kustermann, Gustavo Aguilar, Shinya Matsuda, Ning Zhao, Timothy J. Stasevich, Markus Affolter and Giorgos Pyrowolakis
DOI: 10.1242/dev.191700

Editor: Thomas Lecuit

Review timeline
Original submission: 18 April 2020
Editorial decision: 8 June 2020
First revision received: 14 December 2020
Editorial decision: 13 January 2021
Second revision received: 19 January 2021
Accepted: 9 February 2021

Original submission

First decision letter

MS ID#: DEVELOP/2020/191700

MS TITLE: Protein manipulation using single copies of short peptide tags in cultured cells and in Drosophila melanogaster

AUTHORS: M.Alessandra Vigano, Clara-Maria Ell, Manuela MM Kustermann, Gustavo Aguilar, Shinya Matsuda, Ning Zhao, Timothy J Stasevich, Giorgos Pyrowolakis, and Markus Affolter

Dear Markus,

I have now received all the referees' reports on the above manuscript, and have reached a decision. The referees' comments are appended below, or you can access them online: please go to BenchPress and click on the 'Manuscripts with Decisions' queue in the Author Area.

As you will see, the referees express strong interest in your work, but have some criticisms and recommend a substantial revision of your manuscript before we can consider publication. While not necessarily agreeing with all their comments, I believe all 3 referees make very constructive comments that, if addressed, would increase the potential signficance/impact of the work. I particular, showing in vivo that short peptide tagging of an endogenous protein can be achieved to yield loss of function would be necessary for publication. As Reviewer 1, said this should relatively stratighforward. If you are able to revise the manuscript along the lines suggested, which may involve further experiments, I will be happy receive a revised version of the manuscript. Your revised paper will be re-reviewed by one or more of the original referees, and acceptance of your manuscript will depend on your addressing satisfactorily the reviewers' major concerns. Please also note that Development will normally permit only one round of major revision.

We are aware that you may currently be unable to access the lab to undertake experimental revisions. If it would be helpful, we encourage you to contact us to discuss your revision in greater detail. Please send us a point-by-point response indicating where you are able to address concerns raised (either experimentally or by changes to the text) and where you will not be able to do so

© 2021. Published by The Company of Biologists under the terms of the Creative Commons Attribution License (https://creativecommons.org/licenses/by/4.0/).

within the normal timeframe of a revision. We will then provide further guidance. Please also note that we are happy to extend revision timeframes as necessary.

Please attend to all of the reviewers' comments and ensure that you clearly highlight all changes made in the revised manuscript. Please avoid using 'Tracked changes' in Word files as these are lost in PDF conversion. I should be grateful if you would also provide a point-by-point response detailing how you have dealt with the points raised by the reviewers in the 'Response to Reviewers' box. If you do not agree with any of their criticisms or suggestions please explain clearly why this is so.

Reviewer 1

Advance summary and potential significance to field

Protein binders that can recognize a specific tag linked to a protein of interest (POI) are emerging as powerful tools to manipulate POI in time and space. In this manuscript, Vigano et al. further characterize four short tag epitopes in cell culture and one in vivo in Drosophila and describe their potential use for manipulating POIs. They show that these tags can be recognized by their corresponding protein binders in living cells when present as a single copy in a POI. They also show in vivo that one of the tags can be used for degradation and relocalization of POIs. While these tools are exciting, there are major issues with the paper regarding data presentation, novelty, and scope of the study.

Comments for the author

Major issues:
1. When comparing different tags and antibody binding capabilities, the authors should quantify their data rather than describe them qualitatively. The authors should quantify the intensity of the fluorescent signal, such as amount of fluorescent proteins distributed in the cytoplasm and nucleus or their ratio (through ImageJ or other methods). This would make it easier for readers to evaluate the data.
2. The authors tested four different tags/binder systems, all of them have been previously shown to in various cultured cell systems. Thus, it is not clear what the new findings are. Regarding the ALFA system, the authors provide similar data with previous report (Gotzke et al., 2019). In addition, the authors only provided data for Hela cells. If the authors want to argue that these systems are universal and useful, more cell line data and further protein manipulation (re-localization or degradation of tagged proteins) in vivo should be provided.

Minor comments:
1. For figure 1, are there any linkers between the different elements? Since the linker length and position between tag and protein may significantly affect the binding between tags and protein binders, the linker information should be provided at least in supplementary information.
2.‘On the other hand, protein binders such as scFvs, nanobodies, DARPins, Affibodies,’ .
The authors should provide the references.
3. ‘Our control experiments using baits containing tags that were not supposed to be recognized by the different binders revealed some cross-reactivity between the SunTag and the ALFA systems, mostly with the anti-GCN4 scFv recognizing the ALFA tag rather than the reverse (Suppl. Figure 3, panels of G and J). The similarity of the two tags consists in a stretch of only 3 aa (EEL), but this might be sufficient for low affinity binding.’ - The authors should provide more data to support cross-reactivity between the SunTag and the ALFA systems, at least by using western blot.
4. The yellow font color in Figure 1 is not optimal and should be changed.
5. Salivary Gal4 information is missing in the result and figure legend (Figure 6).
6. The image quality of merged channels in the main figures should be improved.
7. Inconsistent labelling makes the manuscript difficult to read. Please use the consistent labelling styles to describe the protein binders and the tagged proteins with simpler labelling at least in manuscript.
8. Using full plasmid names in the manuscript seems too long and complex to read. Simpler labelling may be needed for better readability since this manuscript deals with many different constructs with different tags.
9. The labeling like “aTag” antibody or “aTag” nanobody needs to be re-labeled as “anti-

© 2021. Published by The Company of Biologists under the terms of the Creative Commons Attribution License (https://creativecommons.org/licenses/by/4.0/).

Tag" antibody or "anti-Tag" nanobody to make the text easier to follow.

Reviewer 2

Advance summary and potential significance to field

Protein manipulation using single copies of short peptide tags in cultured cells and in Drosophila melanogaster

In this manuscript, Vigano and colleagues have assembled a toolkit for protein mislocalization and degradation by taking various short tag epitopes and protein binders and generating fusion proteins. Importantly, they use single copies of the tags in vitro (HeLa) and in vivo (Drosophila). The majority of the manuscript focuses on testing whether binders can be directed to the nucleus, mitochondria, membrane or intermediate filaments in HeLa cells by proteins of interest that contain a single copy of the tag recognized by the binder. The authors then direct GFP fused with a binder recognizing HA to the nucleus of the fat body in flies by using nuclear mCherry-HA as a bait . Finally, the authors swap degradFP for degradHA and provide indirect evidence of its efficacy in an eye overgrowth model where UAS-YkiS168A-HA-GFP is down regulated by expression of degradHA. The introduction and background of the manuscript is well-written and the authors provide sufficient detail throughout.

While the generation and assessment of these constructs is valuable and informative for the field, there are many concerns that would argue against publication of this manuscript in Development.

Comments for the author

Major concerns

· The authors have not generated the binders in this manuscript. They only show that the inclusion of tags as a single copy is sufficient for binding. This is a very incremental novelty.

· Experimental methods suffer from caveats of co-transfection and not being able to gauge the expression of binder and bait by design (This could have been achieved e.g. by using a T2A sequence between binder and the bait that would allow for 1:1 expression of both). Therefore, while the comparison of the constructs in HeLa cells is extensive, it is by no means quantitative. Indeed, there is not any quantification in the manuscript and only qualitative assessment of the effects that are often vague.

· The use of many manipulations that the authors suggest through the use of these binders depend on rates of association and dissociation of the binder to the bait. In other words, showing that the binder can be localized to a specific compartment through the use of a bait does not ensure that it can be used as an enzymatic manipulation tool. In the sole example the authors use one of the binders in an enzymatic manipulation context (DegradHA) but they do not show the direct decrease of expression of ykiS168A-HA-GFP.

· Figure 1 has a lot of redundancies and potentially some errors. It is unclear why some constructs produce a fusion protein that includes the CMV promoter sequence (judged by the orange bar). This is glaringly different in the Moontag with gp41_tag. Can the authors clarify or correct?

· The localization of the binders to the intermediate filaments is not compelling. Can the authors provide more evidence that proteins are directed specifically to Ifs and are not just cytoplasmic?

· By Figure 5, the reader will want a clear indication or at least a comparison of the constructs used. It's not immediately clear which ones are working as predicted to a high degree of efficacy (especially since none are quantified). Can the authors provide a comparison table and qualitatively assess the constructs?

© 2021. Published by The Company of Biologists under the terms of the Creative Commons Attribution License (https://creativecommons.org/licenses/by/4.0/).

· figure 6 does not provide a compelling case on how efficient binders work in vivo. The authors only tested for nuclear localization. The authors would have to provide much more data with different proteins and binders in vivo.

· For Figure 7, it is surprising that GMR>deGradHA, YkiS168A-HA-eGFP (Fig. 7C) is suppressed beyond the basal toxicity of GMR alone (7A). Furthermore, it is unclear how efficient degradHA is as there are no images provided.

· The authors suggest the cross-reactivity of ALFA tag and Suntag may be explained by the EEL motif. This is concerning. Does that mean that the binders may be binding to other unrelated proteins? The specificity of the binders to the baits should be shown by immunoprecipitation of the binder (e.g. using anti-GFP nanobodies) and silver stain/ coomassie stain to show how many proteins come down with the binder. IPs will allow better quantification and comparisons of binders to baits too.

Minor points:

· The color code of Figure1 needs to be changed. The yellow text is almost invisible.

· In Figure 1, why are membrane and intermediate filament constructs combined into one section as they are discussed separately in the results?

· In page 3 “half-live” should be changed to half-life.

· The writing in plasmid construction in materials and methods is confusing. “Briefly, the mitochondrial baits containing an Nterminal anchor sequence from the human CISD1 protein (the first 59 amino acids) fused to the N-terminus of mTFP1, were generated from pcDNA4TO-mito-mCherry- 10xGCN4_v4 (Addgene plasmid 60914 (Tanenbaum et al., 2014)) by substituting the 10xGCN4_v4 with each individual tag,”

In conclusion, the manuscript offers limited novelty and lacks quantification. It would need to address these issues to be published in Development.

<u>Reviewer 3</u>

Advance summary and potential significance to field

Cutting edge experimental genetics requires the ability to manipulate protein function in vivo with high spatial and temporal resolution. To achieve this goal, it is necessary to manipulate protein localisation and/or stability. The authors have championed the use of nanobodies for this aim. At first anti-GFP nanobodies have been used to modulate the activity of GFP fusion protein. Here the authors describe a suite of small tags that can similarly be used. The advantage of such tags is that they might be better tolerated than GFP.
Moreover, the availability of small tags opens the way to multiplexing. Here the authors show that a number of preciously characterised small tags (SunTag, MoonTag, HA tags, ALFA tags) can be used to manipulate protein localisation with cognate nanobodies or scFv. They show this mostly in transfected HeLa cells and with overexpressed protein. In one in vivo experiment, the authors show the relocalisation of a fluorescent protein in the Drosophila salivary gland. As another in vivo experiment, the authors show that they can suppress the phenotype of overexpressing yorkie with their approach.

Comments for the author

Although all the epitope binder pairs have been described elsewhere, the benefit of the present paper is that they are all compared in the same assay. This is potentially a wonderful resource for cell and developmental biologists. The downside of the paper is that it promises a method to interfere with any protein of interest and yet only shows the manipulation of exogenously expressed fluorescent proteins, mostly in HeLa cells. HeLa cells are an excellent system to test out combinations, but one would expect more sophisticated applications to be shown. The power of the

© 2021. Published by The Company of Biologists under the terms of the Creative Commons Attribution License (https://creativecommons.org/licenses/by/4.0/).

approach proposed by the authors is the potential to manipulate endogenously expressed proteins. And yet, no example of this is shown. The examples shown with Drosophila are with overexpressed proteins. This paper would be much stronger if they could illustrate a true loss of function experiment with an endogenously tagged protein. Many mimic lines express HA or FLAG-tagged proteins so it would be relatively straightforward for the authors to illustrate the power of their approach with some of these lines.

First revision

Author response to reviewers' comments

Reviewer 1

Major issues:
1.When comparing different tags and antibody binding capabilities, the authors should quantify their data rather than describe them qualitatively. The authors should quantify the intensity of the fluorescent signal, such as amount of fluorescent proteins distributed in the cytoplasm and nucleus or their ratio (through ImageJ or other methods). This would make it easier for readers to evaluate the data.

The main reason why we opted not to quantify the images in the first version, is that the assay of transient transfection is not quantitative. There are too many variables, linked to transfection efficiency, promoter competition, number of passages of the HeLa cells, time of acquisition of the images after mounting, which could not be tightly controlled for each round of experiments. We were assessing if a single tag is sufficient to bind and locally enrich a free diffusible protein (the binder) in different cell compartments; we were interested in a qualitative assessment and for our type of downstream application (functionalization in vivo) the binding affinity or strength is important but consistency, reproducibility and “flexibility” are more essential.
Nevertheless, we now provide a semi-quantitative analysis on selected images performed with Image J. Two lines encompassing the (sub)structures of interest were drawn on single z stack(s) and the function "Plot profile" in FIJI was applied to visualize the fluorescence intensity signal in all three channels. See attached file.

2.The authors tested four different tags/binder systems, all of them have been previously shown to in various cultured cell systems. Thus, it is not clear what the new findings are. Regarding the ALFA system, the authors provide similar data with previous report (Gotzke et al., 2019). In addition, the authors only provided data for Hela cells. If the
authors want to argue that these systems are universal and useful, more cell line data and further protein manipulation (re-localization or degradation of tagged proteins) in vivo should be provided.

We now provide additional in vivo data to address this point. In addition to the data with overexpressed proteins in Drosophila, we now show that the deGradHA can be used for the manipulation of endogenously tagged proteins carrying single or multiple HA epitope tags. Specifically, we applied genome engineering to generate flies expressing, from its endogenous genomic locus, the BMP receptor Tkv carrying a single C-terminal HA tag followed by eGFP. The generation of such flies was necessary; despite the wide usage of the HA tag, endogenously tagged proteins carrying a single copy of HA are (to our knowledge) not available. We used a second tag (eGFP) to be able to (i) compare the efficacy of the deGradHA tool with the previously described deGradFP tool on the same protein, and (ii) manipulate and detect the protein independently. We additionally tested a version of Tkv carrying three copies of the HA tag at the C-terminus. We show that the deGradHA tool can efficiently affect endogenously tagged proteins as judged by the effects on protein levels and activity. The new data are described in the last paragraph of the Results section of the manuscript and are depicted in the new Fig. 8 and Fig. S11 and S12.

© 2021. Published by The Company of Biologists under the terms of the Creative Commons Attribution License (https://creativecommons.org/licenses/by/4.0/).

Minor comments:
1. For figure 1, are there any linkers between the different elements? Since the linker length and position between tag and protein may significantly affect the binding between tags and protein binders, the linker information should be provided at least in supplementary information.

Yes, there are different linkers in different constructs. The amino acid sequences of the fusion proteins can be provided upon request (as indicated in the Mat and Meth session).

2.'On the other hand, protein binders such as scFvs, nanobodies, DARPins, Affibodies,' . The authors should provide the references.

The references were added

3. 'Our control experiments using baits containing tags that were not supposed to be recognized by the different binders revealed some cross-reactivity between the SunTag and the ALFA systems, mostly with the anti-GCN4 scFv recognizing the ALFA tag rather than the reverse (Suppl. Figure 3, panels of G and J). The similarity of the two tags consists in a stretch of only 3 a a (EEL), but this might be sufficient for low affinity binding.' - The authors should provide more data to support cross-reactivity between the SunTag and the ALFA systems, at least by using western blot.

The cross-reactivity we observed in our assay with anti-GCN4_scFv_GFP and some ALFA containing baits may be a concern when using these two systems at the same time, and we suggested not to use this particular combination. An in-depth analysis of this observed phenomenon is beyond the scope of this report.

4. The yellow font color in Figure 1 is not optimal and should be changed.

We have changed the font color.

5. Salivary Gal4 information is missing in the result and figure legend (Figure 6).

We have added this information in the figure and accompanying manuscript text. We apologize for the omission.

6. The image quality of merged channels in the main figures should be improved.

The quality of the images was not optimal because of the size limitation of the uploaded figures. We ask the editorial board to have less restriction in order to upload figures at high resolution, at least for the main ones.

7. Inconsistent labelling makes the manuscript difficult to read. Please use the consistent labelling styles to describe the protein binders and the tagged proteins with simpler labelling at least in manuscript.

See below.

8. Using full plasmid names in the manuscript seems too long and complex to read. Simpler labelling may be needed for better readability since this manuscript deals with many different constructs with different tags.

See below.

9. The labeling like "aTag" antibody or "aTag" nanobody needs to be re-labeled as "anti-Tag" antibody or "anti-Tag" nanobody to make the text easier to follow.

We fully agree with points 7-9. We tried to simplify and unify all the names in the figures and in this revision (Points 7-9). We also use now consistently the "anti-Tag" term to refer to binders. We hope that now that the manuscript is clearer and apologize for the inconvenience.

© 2021. Published by The Company of Biologists under the terms of the Creative Commons Attribution License (https://creativecommons.org/licenses/by/4.0/).

Reviewer 2:
Major concerns

The authors have not generated the binders in this manuscript. They only show that the inclusion of tags as a single copy is sufficient for binding. This is a very incremental novelty.

We agree that indeed, the binders used in the study have been described before. However, we believe that the strength of the paper relies in the systematic comparison of the performance of these different binders towards proteins carrying a single, short epitope. Many of these binders have only been used to recognize proteins having multiple copies of the short tag, ranging from between 10 to roughly 25 copies. In addition, we generated functionalized versions of some of the binders and demonstrate their activity in vivo.

Experimental methods suffer from caveats of co-transfection and not being able to gauge the expression of binder and bait by design (This could have been achieved e.g. by using a T2A sequence between binder and the bait that would allow for 1:1 expression of both). Therefore, while the comparison of the constructs in HeLa cells is extensive, it is by no means quantitative. Indeed, there is not any quantification in the manuscript and only qualitative assessment of the effects that are often vague.

We agree that the transient co-transfection assay is not quantitative, but we were assessing if a single tag is sufficient to bind and locally attract a free diffusible protein (the binder) in different cell compartments; we were interested in a “quality” assessment and for our type of downstream application (functionalization in vivo) the binding affinity or strength is important but consistency, reproducibility and “flexibility” are more essential.
Nevertheless, we now provide a semi-quantitative analysis on selected images performed with Image J: two lines encompassing the (sub)structures of interest were drawn on single z stack(s) and the function "Plot profile" in FIJI was applied to visualize the fluorescence intensity signal in all the three channels. See attached file.

· The use of many manipulations that the authors suggest through the use of these binders depend on rates of association and dissociation of the binder to the bait. In other words, showing that the binder can be localized to a specific compartment through the use of a bait does not ensure that it can be used as an enzymatic manipulation tool. In the sole example the authors use one of the binders in an enzymatic manipulation context (DegradHA) but they do not show the direct decrease of expression of ykiS168A-HA-GFP.

We have now added data with endogenously epitope tagged proteins and our deGradHA tool. We show that the tool can affect both the levels of the protein and the activity of the protein (see Fig. 8, FigS11 and Fig. S12 and our response to point 2 of Reviewer 1)

· Figure 1 has a lot of redundancies and potentially some errors. It is unclear why some constructs produce a fusion protein that includes the CMV promoter sequence (judged by the orange bar). This is glaringly different in the Moontag with gp41_tag. Can the authors clarify or correct?

We corrected the figure and thank the reviewer for pointing the error.

· The localization of the binders to the intermediate filaments is not compelling. Can the authors provide more evidence that proteins are directed specifically to Ifs and are not just cytoplasmic?

The precise subcellular localization of the baits (especially in overexpression condition) is beyond the scope of this study. The recruitment of binders to the different "structures" visualized by the mCherry fusion protein to intermediate filaments is much clearer in the figures with high resolution. We ask the editors to have less restriction in order to upload figures at high resolution. Upon request, we can also provide some zoomed images of Vimentin-Tag and corresponding binder co-transfection, which show a clearer colocalization.

· By Figure 5, the reader will want a clear indication or at least a comparison of the constructs used. It’s not immediately clear which ones are working as predicted to a high degree of efficacy

© 2021. Published by The Company of Biologists under the terms of the Creative Commons Attribution License (https://creativecommons.org/licenses/by/4.0/).

(especially since none are quantified). Can the authors provide a comparison table and qualitatively assess the constructs?

As suggested in the discussion, we think that all the system tested are sufficient and more or less equivalent for binding to a single copy Tag in vivo. The relative strength and efficacy can only be assessed at a qualitative level in our assay. In different experimental settings and conditions, they might behave differently.

· figure 6 does not provide a compelling case on how efficient binders work in vivo. The authors only tested for nuclear localization. The authors would have to provide much more data with different proteins and binders in vivo.

Following the reviewers' and editor's requests, we now include new data demonstrating degradation and efficient inactivation of a protein expressed from an endogenously tagged gene.

· For Figure 7, it is surprising that GMR>deGradHA, YkiS168A-HA-eGFP (Fig. 7C) is suppressed beyond the basal toxicity of GMR alone (7A). Furthermore, it is unclear how efficient degradHA is as there are no images provided.

We used GRM homozygotes in the previous version of the paper as control flies (indicated by GMR>, in Fig. 7A in the previous version). This was indeed a poor choice as flies carrying two copies of GMR-Gal4 display some basal toxicity which we do not see in flies carrying a single GRM-Gal4 chromosome. We apologize for the confusion. We have corrected this in the present version by using flies with a single GRM-Gal4 chromosome alone or in combination with a UAS-GFP construct. Eyes of either combination are morphologically wild-type and allow a better comparison with flies expressing activated Yki with or without deGradHA with the same driver in a single copy.

· The authors suggest the cross-reactivity of ALFA tag and Suntag may be explained by the EEL motif. This is concerning. Does that mean that the binders may be binding to other unrelated proteins? The specificity of the binders to the baits should be shown by immunoprecipitation of the binder (e.g. using anti-GFP nanobodies) and silver stain/ coomassie stain to show how many proteins come down with the binder. IPs will allow better quantification and comparisons of binders to baits too.

The cross-reactivity we observed in our assay of anti-GCN4_scFv_GFP and some ALFA containing baits may be a concern for using these two systems at the same time, and we suggested not to use this particular combination. A more accurate analysis of this observed phenomenon is beyond the scope of this report. We are only suggesting that the EEL motif, in the particular context of the two epitopes, may explain the observed cross reactivity. As suggested by the reviewer, different type of experiments could be done for resolving this concern, including possible cross-reactivity with endogenous proteins, but they are beyond the scope if this report.

Minor points:

· The color code of Figure1 needs to be changed. The yellow text is almost invisible.

We changed the color of the text.

· In Figure 1, why are membrane and intermediate filament constructs combined into one section as they are discussed separately in the results?

We followed the reviewer suggestion and separated the membrane construct.

· In page 3 "half-live" should be changed to half-life.

We have changed this.

· The writing in plasmid construction in materials and methods is confusing. "Briefly, the mitochondrial baits containing an Nterminal anchor sequence from the human CISD1 protein (the first 59 amino acids) fused to the N-terminus of mTFP1, were generated from pcDNA4TO-mito-

© 2021. Published by The Company of Biologists under the terms of the Creative Commons Attribution License (https://creativecommons.org/licenses/by/4.0/).

mCherry- 10xGCN4_v4 (Addgene plasmid 60914 (Tanenbaum et al., 2014)) by substituting the 10xGCN4_v4 with each individual tag,”

We corrected the text and thank the reviewer for pointing the error.

Reviewer 3
Although all the epitope binder pairs have been described elsewhere, the benefit of the present paper is that they are all compared in the same assay. This is potentially a wonderful resource for cell and developmental biologists. The downside of the paper is that it promises a method to interfere with any protein of interest and yet only shows the manipulation of exogenously expressed fluorescent proteins, mostly in HeLa cells. HeLa cells are an excellent system to test out combinations, but one would expect more sophisticated applications to be shown. The power of the approach proposed by the authors is the potential to manipulate endogenously expressed proteins. And yet, no example of this is shown. The examples shown with Drosophila are with overexpressed proteins. This paper would be much stronger if they could illustrate a true loss of function experiment with an endogenously tagged protein. Many mimic lines express HA or FLAG-tagged proteins so it would be relatively straightforward for the authors to illustrate the power of their approach with some of these lines.

Thank you! We have now added data with endogenously tagged proteins following your suggestion (see also response to Reviewer 1/point 2 and data of Fig.8, Fig.S11 and Fig.S12).

Second decision letter

MS ID#: DEVELOP/2020/191700

MS TITLE: Protein manipulation using single copies of short peptide tags in cultured cells and in Drosophila melanogaster

AUTHORS: M.Alessandra Vigano, Clara-Maria Ell, Manuela MM Kustermann, Gustavo Aguilar, Shinya Matsuda, Ning Zhao, Timothy J Stasevich, George Pyrowolakis, and Markus Affolter

I have now received all the referees reports on the above manuscript, and have reached a decision. The referees' comments are appended below, or you can access them online: please go to BenchPress and click on the 'Manuscripts with Decisions' queue in the Author Area.

The overall evaluation is positive and we would like to publish your manuscript in Development. Before we can proceed further please revise the manuscript by addressing the few minor comments from reviewers.

Reviewer 1

Advance summary and potential significance to field

N/A

Comments for the author

The authors have addressed my concerns overall. Additionally, they have added more in vivo data.

Regarding my previous minor point #1, the protein sequences of the nanobodies should be made available, as in Table S1 of Yamagata and Sanes (PNAS 2018), to make it easier to anyone that use the approach."

© 2021. Published by The Company of Biologists under the terms of the Creative Commons Attribution License (https://creativecommons.org/licenses/by/4.0/).

Reviewer 2

Advance summary and potential significance to field

The revisions answered many of the major concerns and improved the manuscript. I only have minor comments:

Comments for the author

1- In Figure 1, in peptide binder expression vectors panel the schematic of anti-GCN4_scFv_GFP has a small blue arrow after the anti-GCN4-scFv domain. It is unclear to what that corresponds.

2- The levels of tkvHAeGFP, pMad and Sal along DV in different manipulations (No nanobody, DegradHA and DegradFP) should be quantified in figure 8 and in figure S12 and included in the corresponding figures.

Second revision

Author response to reviewers' comments

We thank the reviewers for the meticulous and thorough reviewing of our study with the title “Protein manipulation using single copies of short peptide tags in cultured cells and in Drosophila melanogaster”. We are very pleased about the positive perception of our revised version. In the following, we address the remaining points raised by the reviewers:
Reviewer 1 Comments for the Author:
The authors have addressed my concerns overall. Additionally, they have added more in vivo data. Regarding my previous minor point #1, the protein sequences of the nanobodies should be made available, as in Table S1 of Yamagata and Sanes (PNAS 2018), to make it easier to anyone that use the approach.

We have now added supplementary Table (Table S1) containing protein sequence information of all binder constructs used in this study.

Reviewer 2 Comments for the Author:
1- In Figure 1, in peptide binder expression vectors panel the schematic of anti-GCN4_scFv_GFP has a small blue arrow after the anti-GCN4-scFv domain. It is unclear to what that corresponds.
We apologize for the omission. The blue arrow indeed corresponds to an HA-tag that is present in the construct. We have added this information in Table S1

2- The levels of tkvHAeGFP, pMad and Sal along DV in different manipulations (No nanobody, DegradHA and DegradFP) should be quantified in figure 8 and in figure S12 and included in the corresponding figures.

We now provide quantification of Tkv, pMad and Sal levels in the Fig. 8 and Fig. S12. We have generated intensity plots of the relative fluorescence along the A-P axis of the V-compartment (control) and D-compartment (apgal4-mediated expression of effectors) to better visualize the effects. Following the suggestion of the reviewer, the quantification is included in the single panels of Fig. 8 and Fig. S12.

Third decision letter

MS ID#: DEVELOP/2020/191700

© 2021. Published by The Company of Biologists under the terms of the Creative Commons Attribution License (https://creativecommons.org/licenses/by/4.0/).

MS TITLE: Protein manipulation using single copies of short peptide tags in cultured cells and in Drosophila melanogaster

AUTHORS: M.Alessandra Vigano, Clara-Maria Ell, Manuela MM Kustermann, Gustavo Aguilar, Shinya Matsuda, Ning Zhao, Timothy J Stasevich, Markus Affolter, and Giorgos Pyrowolakis
ARTICLE TYPE: Techniques and Resources Article

I am happy to tell you that your manuscript has been accepted for publication in Development, pending our standard ethics checks.

© 2021. Published by The Company of Biologists under the terms of the Creative Commons Attribution License (https://creativecommons.org/licenses/by/4.0/).
